# Molecular diversity and structural profiling of the EmrD efflux pump in multidrug-resistant uropathogenic *Escherichia coli*

**DOI:** 10.1371/journal.pone.0362259

**Published:** 2026-11-11

**Authors:** Svala H. Mahmood, Iman H. F. Alnaqshabandy

**Affiliations:** Department of Scientific Research Center, College of Science, University of Duhok, Duhok, Kurdistan Region, Iraq; North Carolina State University at Raleigh: North Carolina State University, UNITED STATES OF AMERICA

## Abstract

Uropathogenic *Escherichia coli* (UPEC) presents a significant therapeutic challenge due to increasing multidrug resistance (MDR). While acquired resistance mechanisms are well-documented, the genetic variability of intrinsic efflux pumps like EmrD remains less characterized. This study investigated the genomic diversity and structural conservation of the EmrD transporter in ten clinical MDR isolates from the Kurdistan Region, Iraq. Isolates were screened for the *emrD* and β-lactamase genes, followed by *emrD* sequencing, phylogenetic analysis, and *in silico* protein modeling. Results confirmed the universal presence of *emrD* alongside multiple resistance genes. Phylogenetic analysis identified notable local genetic diversity, including a highly divergent lineage (DUH 11). Protein modeling mapped non-synonymous substitutions to transmembrane helices 6 and 12, regions critical for the “rocker-switch” conformational changes required for substrate translocation. These findings suggest that while EmrD is a stable component of the MDR machinery, localized genomic divergence may potentially influence efflux efficiency among clinical lineages, warranting future functional validation.

## Introduction

Bacterial efflux pumps (EPs) are integral membrane transport proteins that play a crucial role in protecting bacteria against toxic compounds, including many classes of antibiotics. These pumps extrude structurally diverse substrates from the cytoplasm or periplasm into the external environment, thereby lowering intracellular drug concentrations and reducing susceptibility [1–3]. Beyond antimicrobial resistance, EPs are also involved in detoxification of metabolic by-products, maintenance of cellular homeostasis, and interactions with host tissues [4,5]. Given these multifunctional roles, efflux pumps are increasingly recognized as important contributors not only to resistance but also to bacterial adaptation and virulence.

Among multidrug resistance (MDR) efflux systems, several families have been described, including ATP-binding cassette (ABC), resistance–nodulation–division (RND), major facilitator superfamily (MFS), multidrug and toxic compound extrusion (MATE), small multidrug resistance (SMR), and more recently, the proteobacterial antimicrobial compound efflux (PACE) and AbgT families [6,7]. Members of these families share conserved structural motifs and mechanisms of energy coupling, most commonly using the proton motive force to transport substrates against their concentration gradients. The MFS, in particular, is one of the largest and most diverse transporter families, typically organized into two symmetrical bundles of transmembrane helices that facilitate the movement of chemically distinct molecules [8,9]. These pumps serve as critical defense mechanisms, utilizing the electrochemical gradient to drive the efflux of multiple drug classes while simultaneously maintaining bacterial cellular homeostasis [10]. Several high-resolution structures of MFS transporters have revealed conserved sequence motifs and a central cavity that accommodates substrates ranging from hydrophilic solutes to lipophilic antimicrobial agents [11,12].

Within this superfamily, the EmrD efflux pump of *Escherichia coli* (*E. coli*) has been studied as a prototypical MFS multidrug transporter. EmrD was the first antimicrobial carrier of this family for which a crystal structure was resolved, providing critical insights into the architecture of its 12 transmembrane helices and substrate-binding pocket [13]. Functionally, EmrD contributes to resistance against hydrophobic compounds, including uncouplers and antibiotics, by transporting them across the inner membrane [13,14]. Related systems such as EmrAB-TolC form tripartite complexes that span the entire Gram-negative envelope, yet structural and mechanistic details of EmrD and its homologues remain incompletely understood [15]. Despite advances in characterizing MFS efflux pumps, there are still substantial gaps in linking sequence variation to structural changes and functional consequences in clinically relevant bacterial strains.

Uropathogenic *Escherichia coli* (UPEC) is a major cause of urinary tract infection worldwide, responsible for 75% of uncomplicated and 65% of complicated cases, thereby contributing to significant global morbidity and healthcare costs [16]. Increasing resistance to commonly used antibiotics has complicated treatment of UPEC infections and underscores the importance of intrinsic resistance mechanisms, such as efflux pumps. While major efflux pumps like AcrAB-TolC are well-characterized, the *emrD* transporter remains a distinct variable in the *E. coli* resistance repertoire. Evidence from whole-genome sequencing of clinical isolates demonstrates that *emrD* is maintained alongside diverse resistance determinants in strains capable of causing systemic infections [17]. Consequently, characterizing genetic variation in *emrD* across UPEC isolates may provide important insights into the evolutionary dynamics of efflux systems, their contribution to virulence, and their potential as targets for novel therapeutic strategies.

In this study, we characterized sequence variation in the *emrD* efflux pump among ten clinically isolated *E. coli* strains to identify conserved and divergent features that may influence substrate specificity and functional activity. By integrating comparative genomic and structural analyses, we aim to advance understanding of how EmrD contributes to multidrug resistance and pathogenicity in UPEC.

## Materials and methods

### Bacterial strains and DNA samples

A total of ten de-identified *E. coli* DNA samples were obtained in January, 2025 from the archival repository of the Molecular Microbiology Laboratory, College of Science, University of Duhok. These samples originated from clinical *E. coli* isolates recovered during routine diagnostic procedures from patients diagnosed with urinary tract infections (UTIs) at Azadi Teaching Hospital, Duhok, Kurdistan Region, Iraq. In this Study, isolates are operationally defined as UPEC based on clinical source criteria (recovery as significant monocultures ≥10^5^ CFU/mL from symptomatic UTI patients). Species identity was previously confirmed at the molecular level via housekeeping gene PCR and biochemically using the BD Phonex automated microbiology system during a prior master’s research project [18]. The isolates were selected for the current molecular characterization based on their extensive multidrug-resistant (MDR)profiles.

**Ethical Statement:** The authors had no access to information that could identify individual participants during or after data collection. All samples were fully anonymized and identified only by laboratory codes assigned during the original master’s project. The bacteria were isolated as part of routine clinical UTI treatment and diagnosis, not specifically for the purpose of research. Because this study utilized archived bacterial DNA isolates rather than direct human participation or identifiable medical records, the requirement for formal ethical approval was waived according to the institutional guidelines of the University of Duhok.

Antimicrobial susceptibility testing, previously performed using the Kirby-Bauer disk diffusion method according to CLSI guidelines, confirmed that all selected isolates (n = 10) were fully resistan**t** to penicillins (penicillin and amoxicillin,), third-generation cephalosporins (cefotaxime, ceftriaxone, ceftazidime), fluoroquinolones (ciprofloxacin), and folate pathway inhibitors (trimethoprim-sulfamethoxazole). Phenotypic resistance to cloxacillin was also uniformly observed, which was contextualized strictly as an intrinsic, natural property of *E. coli* due to poor outer membrane permeability rather than an acquired resistance trait. These isolates were specifically selected to represent the most challenging clinical phenotypes in the region, serving as a targeted pilot group to characterize the genetic and structural variation of the *emrD* transporter within a high-resistance genomic context**.** This comprehensive resistance profile served as the basis for investigating the underlying genetic mechanisms, specifically the *emrD* efflux pump and β-lactamase genes. Genomic DNA had been extracted using the traditional chloroform–isoamyl alcohol method and stored at –20 °C until use for molecular analysis.

### Molecular detection of β-Lactamase and the *emrD* gene

Stored genomic DNA from ten *E. coli* isolates was used for PCR amplification to detect selected β-lactamase genes (*bla*_*NDM*_, *bla*_*SHV*_, *bla*_*TEM*_, *bla*_*CTX-M-1*_ *and bla*_*CTX-M-9*_) and the multidrug efflux pump gene *emrD*. The primers used for β-lactamase genes detection were obtained from previous studies [19], while the *emrD* target primers were designed in this study; all sequences are natively summarized in Table 1. PCR reactions were carried out in 20 µL total volume containing 10 µL of 2 × PCR Master Mix (Promega, South Korea), each primer at a final concentration of 0.5 µM, 2 µL of DNA template, and nuclease-free water to the final volume. To monitor for reagent contamination and non-specific amplification, a negative non-template control (NTC; replacing DNA template with nuclease-free water) was routinely included in every PCR batch, consistently demonstrating no non-specific amplification. Amplification of β-lactamase genes was performed under the following general thermal profile: initial denaturation at 95 °C for 5 min; 35 cycles of denaturation at 94 °C for 30 s, primer-specific annealing at the temperature listed in Table 1 for 30 s, and extension at 72 °C for 60 s; followed by a final extension at 72 °C for 5 min.

**Table 1:** List of primers used in this study.

Target gene	Primer Sequence (5’→3’)	Amplicon size (bp)	Annealing(℃)	Reference
*bla*_NDM_	F: GGTTTGGCGATCTGGTTTTC R: CGGAATGGCTCACGATC	621	52	[19]
*bla*_SHV_	F: AGCCGCTTGAGCAAATTAAAC R: ATCCCGCAGATAAATCACCAC	713	60	[19]
*bla*_TEM_	F: CATTTCCGTGTCGCCCTTATTC R: CGTTCATCCATAGTTGCCTGAC	800	60	[19]
*bla*_CTX-M-1_	F: AAAAATCACTGCGTCAGTTCAC R: ACAAACCGTTGGTGACGATT	867	55	[19]
*bla*_CTX-M-9_	F: TATTGGGAGTTTGAGATGGT R: TCCTTCAACTCAGCAAAAGT	933	50	[19]
*emrD*	F: CAGATGGCGCAAACCATTTA R: CACAGCACGATCAACAATCC	1076	55	This study

For detection of the *emrD* efflux pump gene, PCR amplification was performed with the following specific program: pre-denaturation at 95 °C for 3 min; 30 cycles of denaturation at 95 °C for 1 min, annealing at 55 °C for 1 min, and extension at 72 °C for 1.5 min; followed by a final extension at 72 °C for 8 min. PCR products were separated by electrophoresis on 1% agarose gel prepared in a 1 × TBE buffer, stained with SYBR safe DNA gel stain, and visualized under UV illumination. A 100-bp DNA ladder (Thermo Fisher Scientific, South Korea) was used as a molecular size marker.

### Sequence processing and alignment

Representative PCR products from the *emrD* gene were submitted to Macrogen Inc. (Seoul, South Korea) for Sanger sequencing using both forward and reverse primers. Raw DNA sequencing data (forward and reverse reads) were obtained for 10 strains of *emrD*. Sequence chromatograms were visually inspected and quality-trimmed to remove low-quality bases at the 5’ and 3’ ends using the Benchling webserver. End-trimming of primer-binding regions trimmed 31 N-terminal amino acids and 50 C-terminal amino acids, leaving residues 32–345 for analysis across all isolates. Sequences were retained only if Phred quality scores exceeded 30 (99.9% accuracy). In addition, to ensure sequence accuracy, the forward and reverse reads for each strain were aligned pairwise. A consensus sequence was generated for each of the 10 samples by resolving discrepancies between the forward and reverse strands. The ten generated consensus sequences along with the reference *emrD* sequence retrieved from GenBank using *E. coli* strain k-12 substrain MG1655 and two prototypical UPEC reference strains—CFT073 (Accession: AAN83032.1) and 536 (Accession: WP_001335494.1) were then subjected to multiple sequence alignment (MSA) using Clustal Omega v1.2.4 [20]. Inclusion of prototypical UPEC reference genomes enabled the explicit differentiation of shared UPEC background polymorphisms from local, isolate-specific substitutions. The alignment was inspected for gaps and conserved regions. Following alignment, Single Nucleotide Polymorphisms (SNPs) were identified by comparing the aligned sequences against a reference strain. The *emrD* gene sequences for all ten clinical isolates were deposited in the NCBI GenBank database under accession numbers (PZ196343, PZ196344, PZ196345, PZ196346, PZ196347, PZ196348, PZ196349, PZ196350, PZ196351, PZ196352).

### Phylogenetic analysis

Phylogenetic relationships were analyzed using MEGA (Molecular Evolutionary Genetics Analysis) software, version 11.0. Prior to tree construction, the “Find Best DNA/Protein Models” feature in MEGA was used to determine the best-fit substitution model for the data. The model with the lowest Bayesian Information Criterion (BIC) score is considered to describe the substitution pattern the best [21]. Hasegawa-Kishino-Yano (HKY) + Gamma (G), was determined as the best fit model and was selected for analysis. A phylogenetic tree was constructed using the Maximum Likelihood (ML) method. Branch support was assessed using a bootstrap analysis with 1000 replicates.

### In Silico conservation and frequency analysis

To evaluate the broader evolutionary conservation of the identified EmrD variants, a comparative analysis was performed using the NCBI Basic Local Alignment Search Tool (BLASTp). The protein sequence of the *E. coli* K-12 EmrD reference was used as a query against the non-redundant protein sequences (nr) database. A curated panel of 25 representative *E. coli* reference sequences displaying high query coverage (≥98%) and sequence identity (≥95%) across diverse pathotypes and geographic origins were retrieved. This non-redundant global dataset was used to calculate the frequency of specific amino acid substitutions (e.g., A173T and V304I) and to determine whether observed mutations occurred within conserved transmembrane helices or variable loop regions.

## Results

### Antibiotic resistance profiles

All ten *E. coli* isolates included in this study exhibited a uniform multidrug-resistant (MDR) phenotype, displaying 100% resistance to amoxicillin, cefotaxime, ceftriaxone, ceftazidime, ciprofloxacin, and trimethoprim-sulfamethoxazole. The observed resistance to third-generation cephalosporins (cefotaxime, ceftazidime) is phenotypically consistent with the production of Extended-Spectrum β-Lactamases (ESBLs), justifying the screening for *bla*_CTX-M_ and *bla*_TEM_ genes. Furthermore, the universal resistance to ciprofloxacin suggests the involvement of active efflux mechanisms or target site mutations, providing the rationale for sequencing the *emrD* multidrug efflux pump gene in these strains.

### PCR amplification of the *emrD* gene

The PCR amplification of the *emrD* gene was successful for all 10 clinical *E. coli* isolates (DUH 01, 02, 03, 04, 05, 06, 09, 10, 11, and 21). Agarose gel electrophoresis revealed clear, single DNA bands of the expected size, 1,076 bp (corresponding to the full-length coding sequence), for all samples. No non-specific amplification or primer dimers were observed, confirming the specificity of the primers used (Fig 1).

**Fig 1:**
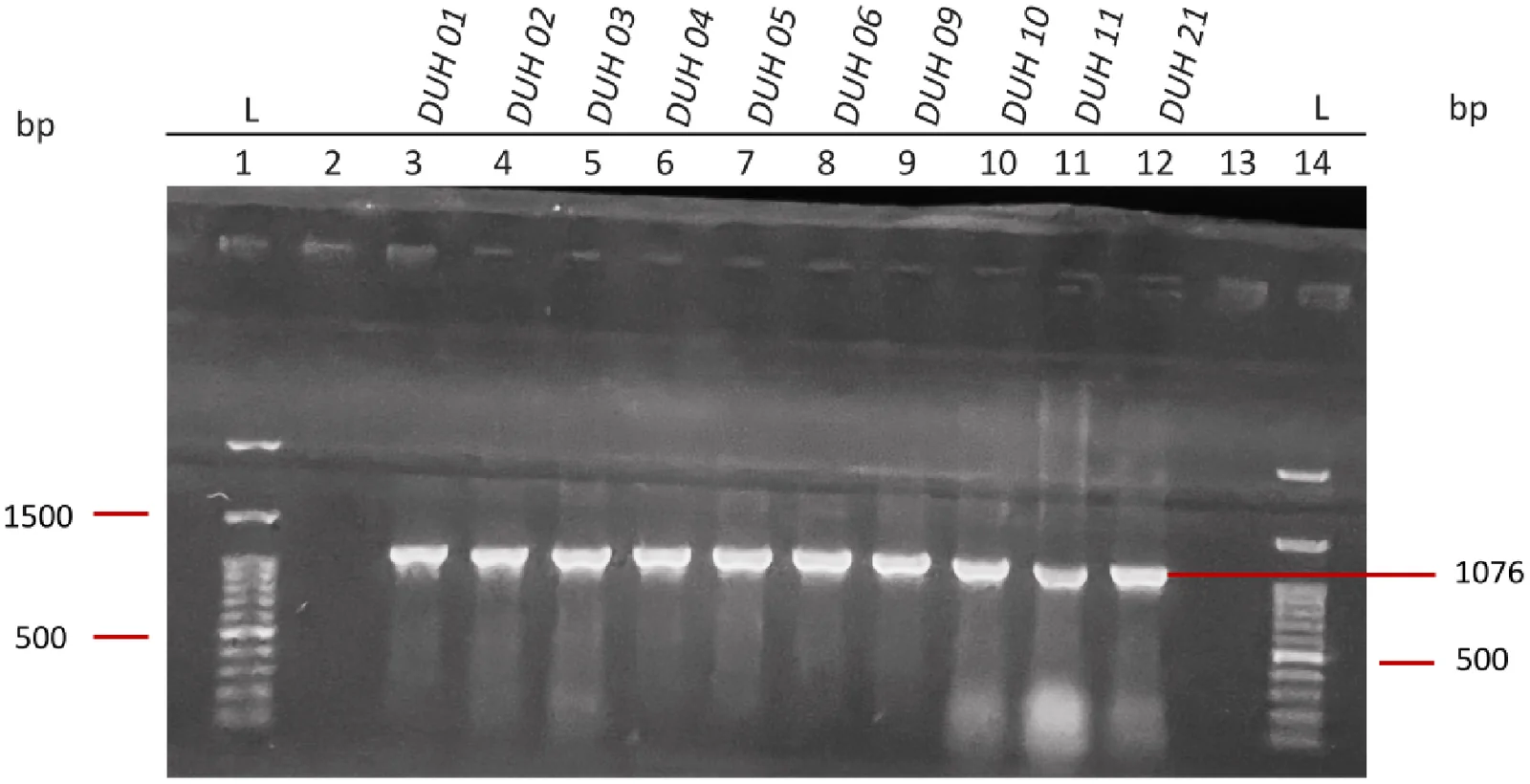
PCR amplification of the *emrD* gene in multidrug-resistant *E. coli* isolates. Representative 1% agarose gel electrophoresis image showing the PCR products of the *emrD* efflux pump gene. **Lane L:** 100 bp DNA Ladder (Thermo Fisher Scientific) serving as the molecular size marker. **Lanes 3–12:** PCR amplicons from ten clinical *E. coli* isolates (DUH 01 through DUH 21). All lanes exhibit a single, distinct band at the expected size of **1,076 bp** (indicated by the red marker), confirming the presence of the *emrD* gene in all tested strains.

The primers used for both β-lactamase and efflux pump detection are detailed in Table 1. The successful amplification allowed for subsequent sequencing and phylogenetic analysis of all collected strains.

### Phylogenetic analysis of EmrD transmembrane protein sequences

The phylogenetic relationship between the 10 sequenced strains (DUH series) and the reference *emrD* strain was analyzed based on the transmembrane protein-encoding DNA consensus sequences. The resulting tree reveals that the strains do not form a single uniform cluster but rather distribute into three distinct clades and one divergent lineage (Fig 2). The first major clade, cluster I, comprises strains that exhibit high sequence conservation with the reference. Strains DUH 09, DUH 05, DUH 06, and DUH 02 clustered tightly with the *emrD* Reference strain. The DUH 09 strain and the *emrD* Reference share a highly similar evolutionary position, with minimal branch lengths of 0.001 and 0.000, respectively. DUH 05 and DUH 06 exhibited zero divergence from their respective internal nodes (branch length 0.000), indicating identical sequences in the analyzed region relative to their immediate ancestors. DUH 02 diverged slightly within this group with a minor branch length of 0.002. A second distinct clade, cluster II, contains strains DUH 04 and DUH 10. This group is separated from the reference clade by a significant branch length of 0.012. Within this cluster, DUH 04 is more conserved (branch length 0.001), whereas DUH 10 shows greater genetic variation (branch length 0.004). The third clade, cluster III, includes strains DUH 03, DUH 21, and DUH 01. DUH 03 and DUH 21 form a closely related pair with minimal divergence (branch lengths 0.001 and 0.000, respectively). DUH 01 branches off earlier from this pair with a branch length of 0.006. Strain DUH 11 was identified as the most genetically distinct isolate in this dataset. It occupies a basal position in the tree with the longest individual terminal branch length of 0.011, suggesting it has accumulated the highest number of nucleotide substitutions relative to the other strains and the reference. Overall Diversity The scale bar indicates a genetic distance of 0.01 nucleotide substitutions per site. The topology suggests that while a subset of the local strains (Cluster I) has remained conserved relative to the reference, significant evolutionary divergence has occurred in the remaining isolates, particularly in DUH 11. However, without a genome-wide phylogenetic framework, it remains unclear whether these variations reflect evolutionary pressures acting specifically on the *emrD* gene or simply represent differences in the broader clonal background among these bacterial lineages.

**Fig 2:**
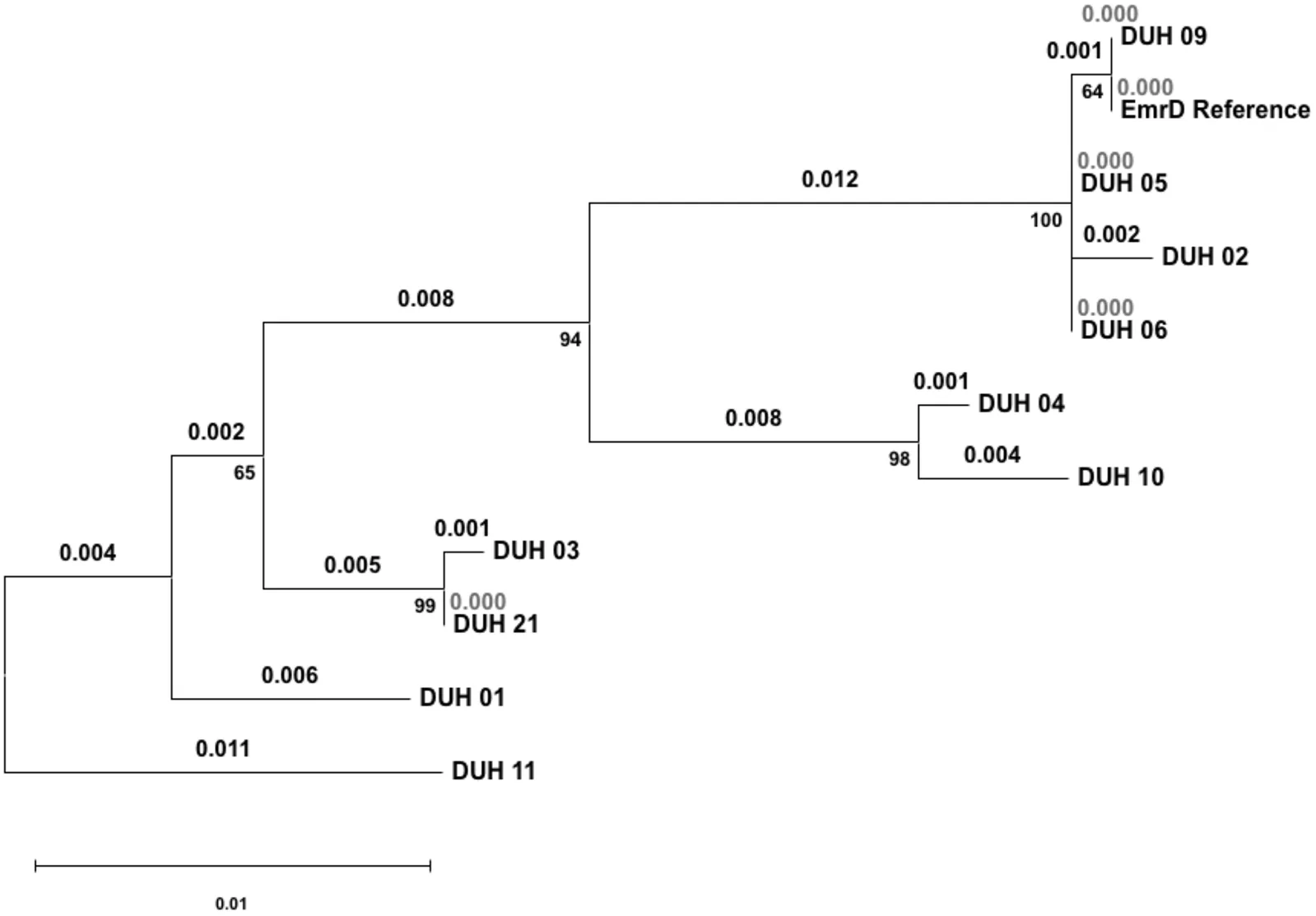
Phylogenetic relationships of *emrD* transmembrane protein sequences. Maximum Likelihood tree constructed using the Hasegawa-Kishino-Yano (HKY) substitution model with Gamma distribution, based on the alignment of *emrD* consensus sequences from 10 clinical isolates (DUH series) and a reference strain. Branch lengths indicate the number of nucleotide substitutions per site (scale bar = 0.01). Numbers at nodes represent bootstrap support values. The tree resolves into three primary clusters: Cluster I (comprising isolates DUH 02, DUH 05, DUH 06, DUH 09, and the reference K-12 MG1655 strain), Cluster II (comprising isolates DUH 04 and DUH 10), Cluster III (comprising isolates DUH 01, DUH 03, and DUH 21), and one divergent, basal outlier (isolate DUH 11).

### Distribution of β-lactamase resistance genes

PCR screening for β-lactamase determinants revealed that all 10 clinical isolates (100%) harbored *bla*_*CTX-M-1*_ and *bla*_*TEM*_ genes. None of the isolates carried *bla*_*NDM*_. The distribution of *bla*_*SHV*_ and *bla*_*CTX-M-9*_ varied among the strains and showed partial congruence with the phylogenetic clustering of *emrD* (Table 2). The *bla*_*SHV*_ gene was detected in 60% (6/10) of the isolates. Notably, both strains in the intermediate phylogenetic clade (DUH 04 and DUH 10) and the cluster III strain DUH 01 were negative for *bla*_*SHV*_. In contrast, the *bla*_*SHV*_ gene was present in the majority of the reference-associated cluster (DUH 02, DUH 05, DUH 06) and the divergent outlier DUH 11. Strain DUH 06 exhibited a unique multidrug-resistance genotype, being the sole isolate to harbor *bla*_*CTX-M-9*_, in addition to *bla*_*CTX-M-1*_, *bla*_*TEM*_, and *bla*_*SHV*_.

**Table 2:** Genotypic profile of β-lactamase resistance genes in clinical isolates. Presence (+) or absence (-) of five major β-lactamase gene families (*bla*_*NDM*_, *bla*_*SHV*_ *bla*_*TEM*_ *bla*_*CTX-M-1*_ *bla*_*CTX-M-9*_*)* detected via PCR. Strains are listed corresponding to their phylogenetic identifiers.

Strain ID	*bla*_NDM_	*bla*_CTX-M-1_	*bla*_CTX-M-9_	*bla*_SHV_	*bla*_TEM_
DUH 01	–	+	–	–	+
DUH 02	–	+	–	+	+
DUH 03	–	+	–	+	+
DUH 04	–	+	–	–	+
DUH 05	–	+	–	+	+
DUH 06	–	+	+	+	+
DUH 09	–	+	–	–	+
DUH 10	–	+	–	–	+
DUH 11	–	+	–	+	+
DUH 21	–	+	–	+	+

### Amino acid substitutions and structural localization in EmrD

Translation of the nucleotide sequences into protein sequences revealed three distinct non-synonymous amino acid substitutions among the study isolates compared to the reference strain (Table 3). Structural mapping of these residues indicated that all observed mutations are located within key transmembrane helices (TMH) of the EmrD transporter (Fig 3). To assess the evolutionary significance of these changes, a comparative analysis was performed against 25 *E. coli* reference sequences (S1 Fig). Two mutations were identified within the sixth transmembrane helix, a region critical for the structural integrity of the MFS transporter core. Conservation mapping showed that while TMH6 is generally a moderately variable (blue-coded) region, specific residues remain highly conserved (S1 Fig). Strain DUH 10 displayed a V170A substitution (Valine to Alanine) in TMH6, which comparative alignment revealed is a baseline polymorphism shared with UPEC reference strain CFT073 (Accession: AAN83032.1). Notably, strain DUH 02 exhibited an A173T substitution (Alanine to Threonine), representing a significant physicochemical shift from a non-polar to a polar residue and standing as a novel variant absent in commensal K-12, UPEC reference strains, and wider BLAST datasets (Table 3). A recurrent substitution was observed in the twelfth transmembrane helix near the C-terminus of the protein. At position 304, Valine was substituted with Isoleucine (V304I) in both Strain DUH 04 and Strain DUH 10 (S2 Fig). While position 304 is located within a strictly conserved (red-coded) region of the *E. coli emrD* consensus (S1 Fig), our analysis revealed significant biochemical plasticity at this site across different strains, including bulky aromatic residues like Phenylalanine (F) and Tryptophan (W). Cross-strain alignment demonstrated that Isoleucine at position 304 (V304I), observed in DUH 04 and DUH 10, is conserved across UPEC reference lineages, including strains CFT073 and 536. While position 304 resides within a conserved region of the consensus, the conservative aliphatic nature of V304I reflects neutral evolutionary divergence characteristic of UPEC isolates rather than a novel destabilizing mutation (Table 3).

**Table 3:** Summary of genomic diversity, evolutionary conservation, and structural impact of *emrD* mutations in clinical *E. coli* isolates.

Sample ID	Phylogenetic Cluster	DNA Mutation (Nucleotide Level)	Amino Acid Change (Protein Level)	Structural Location	UPEC Reference Match	Biochemical Shift	Conservation (*E. coli*)	Variant Status & Frequency (n = 25)*
DUH 01, 03, 05, 06, 09, 21	Cluster I / III	None	None	N/A	Matches K-12, CFT073 & 536	N/A	N/A	Wild-Type Baseline
DUH 02	Cluster I (Variant)	GCT → ACT	Ala173Thr (A173T)	Transmembrane Helix 6	Unique (No match)	Non-polar → Polar	Variable (Blue)	Novel Clinical Variant (4%)
DUH 10	Cluster II	GTT → GCT GTT → ATT	Val170Ala (V170A) Val304Ile (V304I)	Transmembrane Helix6 Transmembrane Helix 12	Matches CFT073 Matches CFT073 & 536	Aliphatic → Aliphatic Aliphatic → Aliphatic	Conserved (Red) Conserved (Red)	UPEC Lineage Feature (4%) UPEC Baseline Feature (8%)
DUH 04	Cluster II	GTT → ATT	Val304Ile (V304I)	Transmembrane Helix 12	Matches CFT073 & 536	Aliphatic → Aliphatic	Conserved (Red)	UPEC Baseline Feature (8%)

*Frequencies (n = 25) are calculated relative to a preliminary dataset of 25 *E. coli* reference sequences retrieved via NCBI BLASTp to provide sequence context rather than global population estimates. Conservation scores are based on NCBI BLASTp multiple sequence alignment (S1 Fig). Reference strain accessions: K-12 MG1655 (NP_416685.1), CFT073 (AAN83032.1), and 536 (WP_001335494.1).

**Fig 3:**
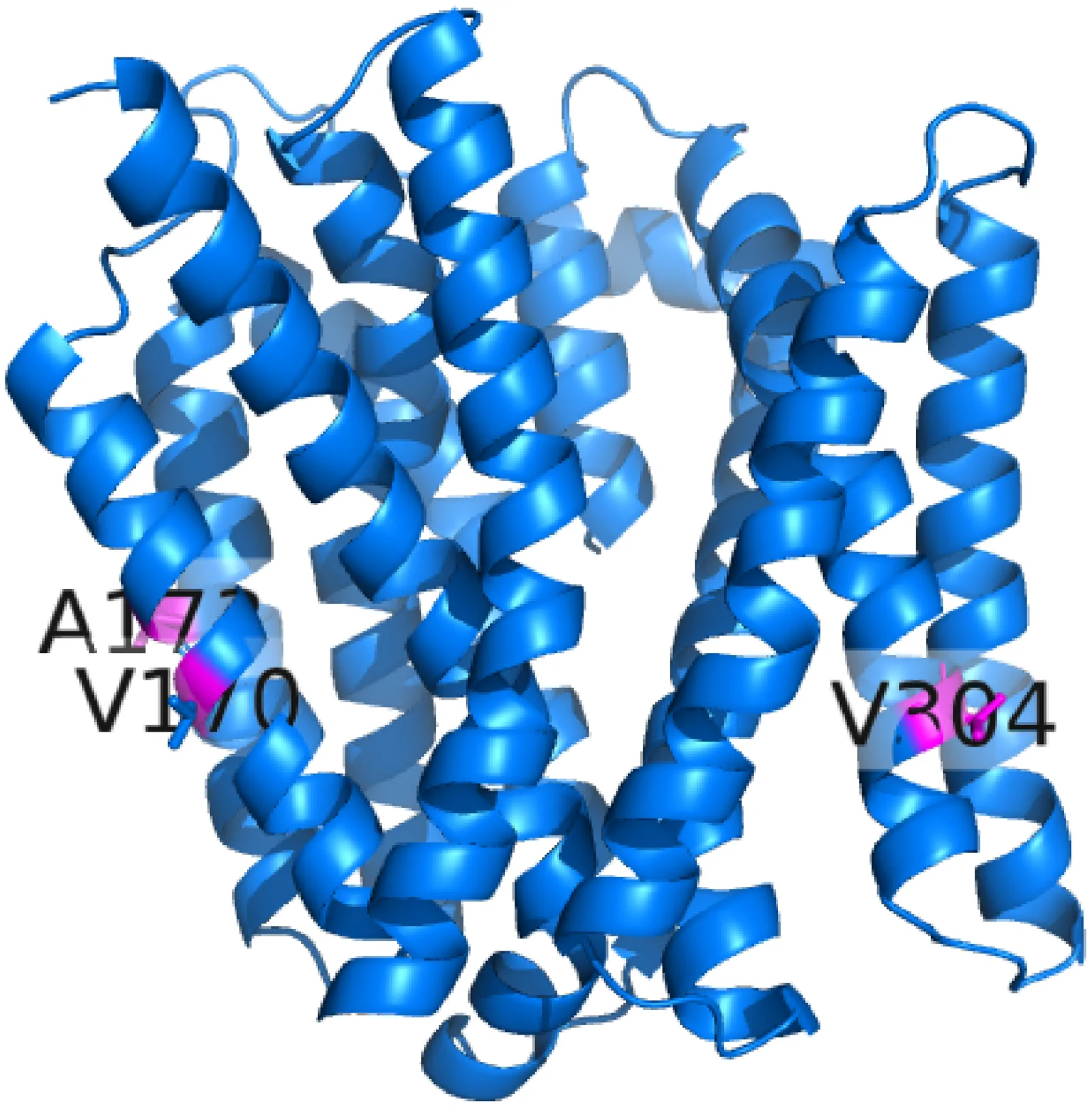
Structural localization of clinical EmrD mutations. Ribbon representation of the EmrD multidrug efflux pump (based on PDB: 2GFP) generated in PyMOL. The three non-synonymous substitutions identified in this study are highlighted in magenta and labeled with their corresponding single-letter amino acid codes. V170 and A173 are situated within Transmembrane Helix 6 (TMH6), while V304 is located within Transmembrane Helix 12 (TMH12). This illustration shows the positions of these variants within the transporter framework, providing a structural context for future studies.

Strain DUH 10 emerged as a unique variant in this dataset, harboring mutations in two distinct transmembrane domains: V170A in Helix 6 and V304I in Helix 12. This specific mutational profile requires further structural and functional characterization to determine its effects on protein conformation and substrate transport.

## Discussion

The rising prevalence of multidrug-resistant (MDR) UPEC in the Kurdistan region necessitates a deeper understanding of the interplay between acquired resistance determinants and intrinsic efflux mechanisms. In this study, we provide a genomic characterization of the *emrD* multidrug efflux transporter in clinical isolates. Our results demonstrate that while the *emrD* gene is universally conserved in these MDR strains, it harbors specific non-synonymous mutations within critical transmembrane domains, suggesting localized evolutionary adaptation.

### Physiological role: virulence and host-pathogen interaction

The detection of *emrD* in 100% of the isolates (n = 10) underscores its status as a core housekeeping gene, distinct from the horizontally acquired *bla*_*CTX-M*_ genes. Alvarez-Ortega et al. have previously noted that while acquired genes provide high-level resistance, intrinsic pumps like EmrD are evolutionarily ancient and essential for cellular homeostasis [5]. However, the role of EmrD likely extends beyond mere detoxification. Pasqua *et al.* recently highlighted that MFS pumps act as mediators of “host-pathogen communication” by extruding host-derived antimicrobial signals, such as bile salts, fatty acids, and prostaglandins [22,23]. Furthermore, Fanelli *et al.* demonstrated that the expression of efflux pumps is critical for *E. coli* survival within the oxidative and acidic environment of macrophages [24]. However, because *emrD* is a conserved core gene found in both pathogenic and non-pathogenic *E. coli*, its universal presence likely reflects genomic conservation rather than a specific role in UPEC virulence or host colonization..

### Co-resistance of EmrD variants and β-lactamase genes

The clinical severity of these isolates is highlighted by the co-occurrence of EmrD with *bla*_*CTX-M-1*_ and *bla*_*TEM*_ β-lactamases. This aligns with the “crossroad” hypothesis proposed by Alcalde-Rico *et al.,* where efflux pumps and enzymatic resistance mechanisms evolve cooperatively to ensure survival against combined therapies [3]. Such co-selection suggests that EmrD variants are maintained alongside β-lactamase genes in Iraqi UPEC isolates. Previous studies have linked efflux pump regulation with biofilm formation and motility [25]

### Phylogenetic diversity and evolutionary dynamics

While efflux pump genes are traditionally viewed as highly conserved elements of the bacterial core genome [4], our phylogenetic analysis reveals unexpected genetic heterogeneity within the *emrD* sequences. The segregation of isolates into three distinct clusters (I, II, and III) indicates that the *emrD* gene in this geographical region is not monoclonal but rather associated with diverse *E. coli* lineages (Fig 2). The predominance of Cluster I (containing 60% of isolates, including isolate DUH 06) suggests the local expansion of a dominant UPEC clone that maintains the wild-type *emrD* sequence. In contrast, the extensive branch length observed for strain DUH 11 highlights a divergent evolutionary trajectory. This outlier status likely reflects the accumulation of silent mutations or a distinct phylogenetic background (e.g., a different *E. coli* phylogroup), similar to the chromosomal heterogeneity recently described in UPEC isolates from Uganda by Maghembe et al. (2024) [17]. We emphasize that the *bla*_*SHV*_-positive status of DUH 11 (along with *bla*_*CTX-M-1*_ and *bla*_*TEM*_) is plasmid-borne and uncoupled from its chromosomal sequence divergence. Importantly, comparative sequence alignment revealed that the V304I substitution is conserved in prototypical UPEC reference strains CFT073 and 536, while V170A matches strain CFT073. Without *dN/dS* analysis or population-scale genomic data, these substitutions reflect neutral lineage-specific polymorphisms within UPEC rather than adaptive evolution under novel selection pressures.

### Structural implications of the EmrD mutations

A pivotal finding of this study is the identification of the V170A mutation in Helix 6 (TM6) and the V304I mutation in Helix 12 (TM12) (Fig 3). Crucially, cross-strain comparisons demonstrate that V304I represents a baseline UPEC lineage feature shared with both reference strains CFT073 and 536, while V170A is shared specifically with strain CFT073. In contrast, the A173T substitution in strain DUH 02 represents a novel, unique clinical variant absent from all reference strains. Structural crystallographic studies of EmrD by Yin *et al.* revealed that the transporter is composed of 12 transmembrane helices surrounding a hydrophobic internal cavity, utilizing a “rocker-switch” mechanism to translocate amphipathic substrates across the inner membrane [13]. This scaffold operates via a ‘rocker-switch’ mechanism, characterized by large-scale alternating access movements between inward-open and outward-open states [26]. Our identification of mutations in TMH6 and TMH12 is significant because these helices are central to this pivoting motion, potentially influencing the speed or efficiency of the switch during substrate export. Ranaweera *et al.* further established that in the MFS, Helices 6 and 12 frequently line the substrate-translocation pathway and contain residues critical for substrate recognition [14]. We hypothesize that the V170A and V304I substitutions modulate the conformational plasticity of the transporter. Recent work by Kumar *et al.* describes MFS binding pockets as “promiscuous” and highly flexible, relying on an “induced fit” mechanism to accommodate diverse antibiotics [12]. The localization of mutations V170A, A173T, and V304I within these central helices is highly significant, as structural flexibility in these regions is required for substrate binding and release. While baseline UPEC lineage polymorphisms (V170A and V304I) were present in 4% and 8% of our initial screening BLAST dataset, respectively, the novel A173T variant remains unobserved across global reference datasets, highlighting its unique distribution in clinical isolate DUH 02. However, their evolutionary significance cannot be determined from sequence data alone and requires further functional and population-level investigation. The substitution of Valine with Alanine (V170A) shared with strain CFT073 reduces side-chain volume, which may alter the packing density of Helix 6, potentially modifying the “structural wobble” required for the transporter to capture newer generation fluoroquinolones or cephalosporins. This is consistent with the possibility that EmrD variants may undergo structural diversification under antibiotic selection pressures, although this remains speculative. Furthermore, the shift from bulky aromatic residues (like F304) to smaller aliphatic residues (like I304) in our isolates and UPEC reference strains is hypothesized to alter the internal cavity volume. Notably, the introduction of a polar Threonine at position 173 in the novel DUH 02 variant could theoretically facilitate new hydrogen-bonding interactions within TMH6 during the conformational cycle. Although these substitutions could theoretically influence the induced-fit mechanism described for MFS transporters, their effects on transporter dynamics and substrate recognition remain unknown without molecular dynamics simulations and functional validation. Therefore, the structural and evolutionary significance of the Cluster II and the novel A173T mutation variants remains unresolved and should be regarded as a hypothesis requiring further structural and functional investigation.

## Limitations

This study has limitations inherent to its pilot nature. First, the sample size (n = 10) limits the generalization of the specific mutation frequencies to the broader Middle Eastern UPEC population. Second, while we identified non-synonymous amino acid substitutions (the novel A173T variant alongside UPEC lineage features V170A and V304I), this study relied on genomic and structural prediction and did not employ molecular dynamics simulations, RT-qPCR to quantify *emrD* transcript levels or site-directed mutagenesis to kinetically validate the V170A/V304I variants. Future studies will focus on the functional characterization of the novel A173T mutant to definitively correlate the genotype with altered minimum inhibitory concentrations (MICs). Third, the scope was bounded by clinical operational categorization of UPEC rather than molecular virulence or Multi Locus Sequence Typing (MLST)/phylogroup profiling, and β-lactamase genes were characterized by PCR screen alone. Finally, primer end-trimming restricted analyzed sequence coverage to residues 32–345, leaving potential variation in the first 31 N-terminal and last 50 C-terminal amino acids unevaluated.

## Conclusion

In conclusion, this study confirms that *emrD* is a highly conserved yet genetically diverse transporter in clinical UPEC isolates. The identification of a novel structural mutation in TMH6 (A173T in strain DUH 02), alongside baseline UPEC lineage polymorphisms (V170A and V304I), supports a model of “induced fit” evolution in MFS transporters, wherein localized structural variations optimize transport dynamics without compromising overall scaffold stability. While functional assays are required for confirmation, these structural alterations are predicted to potentially influence the pump’s conformational dynamics. These findings, coupled with the presence of ESBL genes, suggest that *emrD* sequence variation represents an active component of the UPEC genomic repertoire, with the potential to play a role in antibiotic tolerance or environmental survival through these observed structural variations.

## Supporting information

S1 Fig Multiple sequence alignment of EmrD from 25 representative *Escherichia coli* reference strains. This alignment was performed using BLASTp to assess the evolutionary conservation of residues at positions 170, 173, and 304. Amino acid conservation is indicated by color coding: red denotes strictly conserved residues, while blue indicates regions of moderate to high variability. The alignment illustrates the rarity of the A173T and V304I substitutions identified in the clinical isolates of the current study.
(RTF)

S1 File Original, uncropped agarose gel electrophoresis image for PCR amplification products. (PNG)

S2 Fig Multiple sequence alignment: The amino acid sequences of ten clinical *E. coli* isolates (DUH 01 to DUH 21) are aligned against the *E. coli* K-12 reference sequence (EmrD_Ref) and two prototypical uropathogenic *E. coli* (UPEC) reference strains: CFT073 (Accession: AAN83032.1) and 536 (Accession: WP_001335494.1). Asterisks (*) indicate positions with fully conserved residues; colons (:) indicate conservation between groups of strongly similar properties; and periods (.) indicate conservation between groups of weakly similar properties. Non-synonymous substitutions are highlighted by a lack of an asterisk at the corresponding position: the novel A173T in strain DUH 02 (TMH6), alongside baseline UPEC lineage-conserved features V170A in strain DUH 10 (TMH6; shared with strain CFT073)), and V304I in strains DUH 04 and DUH 10 (TMH12; shared with strains CFT073 and 536). Note: Terminal primer end-trimming restricted the analyzed clinical sequence range to residues 32–345, leaving The initial 31 amino acids for clinical isolates un-evaluated, while the core transmembrane machinery remains intact and fully aligned.
(PDF)
