# Supplementary material for: Molecular diversity and structural profiling of the EmrD efflux pump in multidrug-resistant uropathogenic Escherichia coli
Source: PLoS One. 2026 Nov 11;21(11):e0362259. doi: 10.1371/journal.pone.0362259 (PMC13754136; doi:10.1371/journal.pone.0362259)
Supplement: Supplementary file 1 [file pone.0362259.s003.pdf]

```
EmrD_Reference      MKRQRNVNLLLMLVLLVAVGQMAQTIYIPAIADMARDLNVREGAVQSVMGAYLLTYGVSQ    60
CFT073_UPEC         MKRQRNVNLLLMLVLLVAVGQMAQTIYIPAIADMARDLNVREGAVQSVMGAYLLTYGVSQ    60
536_UPEC            MKRQRNVNLLLMLVLLVAVGQMAQTIYIPAIADMARDLNVREGAVQSVMGAYLLTYGVSQ    60
DUH 01              -------------------------------ADMARDLNVREGAVQSVMGAYLLTYGVSQ    29
DUH 02              -------------------------------ADMARDLNVREGAVQSVMGAYLLTYGVSQ    29
DUH 03              -------------------------------ADMARDLNVREGAVQSVMGAYLLTYGVSQ    29
DUH 04              -------------------------------ADMARDLNVREGAVQSVMGAYLLTYGVSQ    29
DUH 05              -------------------------------ADMARDLNVREGAVQSVMGAYLLTYGVSQ    29
DUH 06              -------------------------------ADMARDLNVREGAVQSVMGAYLLTYGVSQ    29
DUH 09              -------------------------------ADMARDLNVREGAVQSVMGAYLLTYGVSQ    29
DUH 10              -------------------------------ADMARDLNVREGAVQSVMGAYLLTYGVSQ    29
DUH 11              -------------------------------ADMARDLNVREGAVQSVMGAYLLTYGVSQ    29
DUH 21              -------------------------------ADMARDLNVREGAVQSVMGAYLLTYGVSQ    29
                                                   *****************************

EmrD_Reference      LFYGPISDRVGRRPVILVGMSIFMLATLVAVTTSSLTVLIAASAMQGMGTGVGGVMARTL    120
CFT073_UPEC         LFYGPISDRVGRRPVILVGMSIFMLATLVAVTTSSLTVLIAASAMQGMGTGVGGVMARTL    120
536_UPEC            LFYGPISDRVGRRPVILVGMSIFMLATLVAVTTSSLTVLIAASAMQGMGTGVGGVMARTL    120
DUH 01              LFYGPISDRVGRRPVILVGMSIFMLATLVAVTTSSLTVLIAASAMQGMGTGVGGVMARTL    89
```

```
DUH 02          LFYGPISDRVGRRPVILVGMSIFMLATLVAVTTSSLTVLIAASAMQGMGTGVGGVMARTL    89
DUH 03          LFYGPISDRVGRRPVILVGMSIFMLATLVAVTTSSLTVLIAASAMQGMGTGVGGVMARTL    89
DUH 04          LFYGPISDRVGRRPVILVGMSIFMLATLVAVTTSSLTVLIAASAMQGMGTGVGGVMARTL    89
DUH 05          LFYGPISDRVGRRPVILVGMSIFMLATLVAVTTSSLTVLIAASAMQGMGTGVGGVMARTL    89
DUH 06          LFYGPISDRVGRRPVILVGMSIFMLATLVAVTTSSLTVLIAASAMQGMGTGVGGVMARTL    89
DUH 09          LFYGPISDRVGRRPVILVGMSIFMLATLVAVTTSSLTVLIAASAMQGMGTGVGGVMARTL    89
DUH 10          LFYGPISDRVGRRPVILVGMSIFMLATLVAVTTSSLTVLIAASAMQGMGTGVGGVMARTL    89
DUH 11          LFYGPISDRVGRRPVILVGMSIFMLATLVAVTTSSLTVLIAASAMQGMGTGVGGVMARTL    89
DUH 21          LFYGPISDRVGRRPVILVGMSIFMLATLVAVTTSSLTVLIAASAMQGMGTGVGGVMARTL    89
                ************************************************************

EmrD_Reference  PRDLYERTQLRHANSLLNMGILVSPLLAPLIGGLLDTMWNWRACYLFLLVLCAGVTFSMA    180
CFT073_UPEC     PRDLYERTQLRHANSLLNMGILVSPLLAPLIGGLLDTMWNWRACYLFLLALCAGVTFSMA    180
536_UPEC        PRDLYERTQLRHANSLLNMGILVSPLLAPLIGGLLDTMWNWRACYLFLLVLCAGVTFSMA    180
DUH 01          PRDLYERTQLRHANSLLNMGILVSPLLAPLIGGLLDTMWNWRACYLFLLVLCAGVTFSMA    149
DUH 02          PRDLYERTQLRHANSLLNMGILVSPLLAPLIGGLLDTMWNWRACYLFLLVLCTGVTFSMA    149
DUH 03          PRDLYERTQLRHANSLLNMGILVSPLLAPLIGGLLDTMWNWRACYLFLLVLCAGVTFSMA    149
DUH 04          PRDLYERTQLRHANSLLNMGILVSPLLAPLIGGLLDTMWNWRACYLFLLVLCAGVTFSMA    149
DUH 05          PRDLYERTQLRHANSLLNMGILVSPLLAPLIGGLLDTMWNWRACYLFLLVLCAGVTFSMA    149
DUH 06          PRDLYERTQLRHANSLLNMGILVSPLLAPLIGGLLDTMWNWRACYLFLLVLCAGVTFSMA    149
```

```
DUH 09          PRDLYERTQLRHANSLLNMGILVSPLLAPLIGGLLDTMWNWRACYLFLLVLCAGVTFSMA    149
DUH 10          PRDLYERTQLRHANSLLNMGILVSPLLAPLIGGLLDTMWNWRACYLFLLALCAGVTFSMA    149
DUH 11          PRDLYERTQLRHANSLLNMGILVSPLLAPLIGGLLDTMWNWRACYLFLLVLCAGVTFSMA    149
DUH 21          PRDLYERTQLRHANSLLNMGILVSPLLAPLIGGLLDTMWNWRACYLFLLVLCAGVTFSMA    149
                *************************************************:**.****** 

EmrD_Reference  RWMPETRPVDAPRTRLLTSYKTLFGNSGFNCYLLMLIGGLAGIAAFEACSGVLMGAVLGL    240
CFT073_UPEC     RWMPETRPVDAPRTRLLTSYKTLFGNSGFNCYLLMLIGGLAGIAAFEACSGVLMGAVLGL    240
536_UPEC        RWMPETRPVDAPRTRLLTSYKTLFGNSGFNCYLLMLIGGLAGIAAFEACSGVLMGAVLGL    240
DUH_01          RWMPETRPVDAPRTRLLTSYKTLFGNSGFNCYLLMLIGGLAGIAAFEACSGVLMGAVLGL    209
DUH_02          RWMPETRPVDAPRTRLLTSYKTLFGNSGFNCYLLMLIGGLAGIAAFEACSGVLMGAVLGL    209
DUH_03          RWMPETRPVDAPRTRLLTSYKTLFGNSGFNCYLLMLIGGLAGIAAFEACSGVLMGAVLGL    209
DUH_04          RWMPETRPVDAPRTRLLTSYKTLFGNSGFNCYLLMLIGGLAGIAAFEACSGVLMGAVLGL    209
DUH_05          RWMPETRPVDAPRTRLLTSYKTLFGNSGFNCYLLMLIGGLAGIAAFEACSGVLMGAVLGL    209
DUH_06          RWMPETRPVDAPRTRLLTSYKTLFGNSGFNCYLLMLIGGLAGIAAFEACSGVLMGAVLGL    209
DUH_09          RWMPETRPVDAPRTRLLTSYKTLFGNSGFNCYLLMLIGGLAGIAAFEACSGVLMGAVLGL    209
DUH_10          RWMPETRPVDAPRTRLLTSYKTLFGNSGFNCYLLMLIGGLAGIAAFEACSGVLMGAVLGL    209
DUH_11          RWMPETRPVDAPRTRLLTSYKTLFGNSGFNCYLLMLIGGLAGIAAFEACSGVLMGAVLGL    209
DUH_21          RWMPETRPVDAPRTRLLTSYKTLFGNSGFNCYLLMLIGGLAGIAAFEACSGVLMGAVLGL    209
                ************************************************************
```

```
EmrD_Reference      SSMTVSILFILPIPAAFFGAWFAGRPNKRFSTLMWQSVICCLLAGLLMWIPDWFGVMNVW    300
CFT073_UPEC         SSMTVSILFILPIPAAFFGAWFAGRPNKRFSTLMWQSVICCLLAGLLMWIPDWFGVMNVW    300
536_UPEC            SSMTVSILFILPIPAAFFGAWFAGRPNKRFSTLMWQSVICCLLAGLLMWIPDWFGVMNVW    300
DUH_01              SSMTVSILFILPIPAAFFGAWFAGRPNKRFSTLMWQSVICCLLAGLLMWIPDWFGVMNVW    269
DUH_02              SSMTVSILFILPIPAAFFGAWFAGRPNKRFSTLMWQSVICCLLAGLLMWIPDWFGVMNVW    269
DUH_03              SSMTVSILFILPIPAAFFGAWFAGRPNKRFSTLMWQSVICCLLAGLLMWIPDWFGVMNVW    269
DUH_04              SSMTVSILFILPIPAAFFGAWFAGRPNKRFSTLMWQSVICCLLAGLLMWIPDWFGVMNVW    269
DUH_05              SSMTVSILFILPIPAAFFGAWFAGRPNKRFSTLMWQSVICCLLAGLLMWIPDWFGVMNVW    269
DUH_06              SSMTVSILFILPIPAAFFGAWFAGRPNKRFSTLMWQSVICCLLAGLLMWIPDWFGVMNVW    269
DUH_09              SSMTVSILFILPIPAAFFGAWFAGRPNKRFSTLMWQSVICCLLAGLLMWIPDWFGVMNVW    269
DUH_10              SSMTVSILFILPIPAAFFGAWFAGRPNKRFSTLMWQSVICCLLAGLLMWIPDWFGVMNVW    269
DUH_11              SSMTVSILFILPIPAAFFGAWFAGRPNKRFSTLMWQSVICCLLAGLLMWIPDWFGVMNVW    269
DUH_21              SSMTVSILFILPIPAAFFGAWFAGRPNKRFSTLMWQSVICCLLAGLLMWIPDWFGVMNVW    269
                    ************************************************************


EmrD_Reference      TLLVPAALFFFGAGMLFPLATSGAMEPFPFLAGTAGALVGGLQNI 345
CFT073_UPEC         TLLVPAALFFFGAGMLFPLATSGAMEPFPFLAGTAGALVGGLQNI 345
536_UPEC            TLLIPAALFFFGAGMLFPLATSGAMEPFPFLAGTAGALVGGLQNI 345
DUH_01              TLLVPAALFFFGAGMLFPLATSGAMEPFPFLAGTAGALVGGLQNI 314
```

```
DUH_02          TLLVPAALFFFGAGMLFPLATSGAMEPFPFLAGTAGALVGGLQNI 314
DUH_03          TLLVPAALFFFGAGMLFPLATSGAMEPFPFLAGTAGALVGGLQNI 314
DUH_04          TLLIPAALFFFGAGMLFPLATSGAMEPFPFLAGTAGALVGGLQNI 314
DUH_05          TLLVPAALFFFGAGMLFPLATSGAMEPFPFLAGTAGALVGGLQNI 314
DUH_06          TLLVPAALFFFGAGMLFPLATSGAMEPFPFLAGTAGALVGGLQNI 314
DUH_09          TLLVPAALFFFGAGMLFPLATSGAMEPFPFLAGTAGALVGGLQNI 314
DUH_10          TLLIPAALFFFGAGMLFPLATSGAMEPFPFLAGTAGALVGGLQNI 314
DUH_11          TLLVPAALFFFGAGMLFPLATSGAMEPFPFLAGTAGALVGGLQNI 314
DUH_21          TLLVPAALFFFGAGMLFPLATSGAMEPFPFLAGTAGALVGGLQNI 314
                ***:*****************************************
```
