# Supplementary material for: Molecular diversity and structural profiling of the EmrD efflux pump in multidrug-resistant uropathogenic Escherichia coli
Source: PLoS One. 2026 Nov 11;21(11):e0362259. doi: 10.1371/journal.pone.0362259 (PMC13754136; doi:10.1371/journal.pone.0362259)
Supplement: Supplementary file 2 [file pone.0362259.s001.rtf]

Query_EmrD     1        -   -   -   ------------------------ADMARDL---NVREGAVQSVMGAYLLTYGVSQLFYGPISD  37

P31442.2       1        M   K   R[5]LLLMLVLLVA-VGQMAQTIYIPAIADMARDL---NVREGAVQSVMGAYLLTYGVSQLFYGPISD  68

P37597.3       1        M   -   -[5]FLVWLAGLSV-LGFLATDMYLPAFAAIQADL---QTPASAVSASLSLFLAGFAAAQLLWGPLSD  66

B7NF27.1       1        M   S   R   FLICSFALVL-LYPAGIDMYLVGLPRIAADL---NASEAQLHIAFSVYLAGMAAAMLFAGKVAD  63

B1LL36.1       1        M   S   R   FLICSFALVL-LYPAGIDMYLVGLPRIAADL---NASEAQLHIAFSVYLAGMAAAMLFAGKVAD  63

B7NR12.1       1        M   S   R   FLICSFALVL-LYPAGIDMYLVGLPRIAADL---NASEAQLHIAFSVYLAGMAAAMLFAGKVAD  63

A7ZTR5.1       1        M   S   R   FLICSFALVL-LYPAGIDMYLVGLPRIAADL---NASEAQLHIAFSVYLAGMAAAMLFAGKVAD  63

B1X9T9.1       1        M   S   R   FLICSFALVL-LYPAGIDMYLVGLPRIAADL---NASEAQLHIAFSVYLAGMAAAMLFAGKVAD  63

A8A6H2.1       1        M   S   R   FLICSFALVL-LYPAGIDMYLVGLPRIAADL---NASEAQLHIAFSVYLAGMAAAMLFAGKVAD  63

P28246.4       1        M   T   T[7]IVFILGLLAM-LMPLSIDMYLPALPVISAQF---GVPAGSTQMTLSTYILGFALGQLIYGPMAD  70

Q0TAZ8.1       1        M   S   R   FLICSFALVL-LYPAGIDMYLVGLPRIAADL---NASEAQLHIAFSVYLAGMAAAMLFAGKVAD  63

B7UMH7.1       1        M   S   R   FLICSFALVL-LYPAGIDMYLVGLPRIAADL---NASEAQLHIAFSVYLAGMAAAMLFAGKVAD  63

B7N2F5.1       1        M   S   R   FLICSFALVL-LYPAGIDMYLVGLPRIAADL---NASEAQLHIAFSVYLAGMAAAMLFAGKVAD  63

A1AHP3.1       1        M   S   R   FLICSFALVL-LYPAGIDMYLVGLPRIAADL---NASEAQLHIAFSVYLAGMAAAMLFAGKVAD  63

B5YXB4.1       1        M   S   R   FLICSFALVL-LYPAGIDMYLVGLPRIAADL---NASEAQLHIAFSVYLAGMAAAMLFAGKVAD  63

P39386.1       1        M[4]T   R[6]FPMALILYDF-AAYLSTDLIQPGIINVVRDF---NADVSLAPAAVSLYLAGGMALQWLLGPLSD  73

Q8XB84.1       1        M[4]A   R[6]FPMALILYDF-AAYLSTDLIQPGIINVVRDF---NADVSLAPAAVSLYLAGGMALQWLLGPLSD  73

P0AEY8.1       1        M[7]A   R[7]FPLCLVLYEF-STYIGNDMIQPGMLAVVEQY---QAGIDWVPTSMTAYLAGGMFLQWLLGPLSD  77

P31474.1       1        M[6]S[3]-   -LPWIAAMAFfMQALDATILNTALPAIAHSL---NRSPLAMQSAIISYTLTVAMLIPVSGWLAD  71

P77726.2       1        M[7]G[1]R[2]-TWGLGTVFS-LRMLGMFMVLPVLTTYGMAL--qGASEALIGIAIGIYGLTQAVFQIPFGLLSD  73

P43531.2       1    [14]T[7]P[5]R[7]VTLALFSAGL-ATFALLYCVQPILPVLSQEF---GLTPANSSISLSISTAMLAIGLLFTGPLSD  96

P76269.2       1        M[6]G[5]R[6]IVIGISMAVL-----DGAIANVALPTIATDL---HATPASSIWVVNAYQIAIVISLLSFSFLGD  76

P0AEJ0.1       1        M[4]P   L[7]MTIALSLATF-MQVLDSTIANVAIPTIAGNL---GSSLSQGTWVITSFGVANAISIPLTGWLAK  74

P02981.1       1        M[1]S   N[2]-LIVILGTVT-LDAVGIGLVMPVLPGLLRDIvhsDSIASHYGVLLALYALMQFLCAPVLGALSD  68

P02982.2       1        M[1]P   N[2]-LIVILSTVA-LDAVGIGLIMPVLPGLLRDLvhsNDVTAHYGILLALYALMQFACAPVLGALSD  68


Query_EmrD     38   RVGRRPVILVGMSIFMLATLVAVTTSSLTVLIAASAMQGMGTGVGGVMARTLPRDLYERTQLRHANSLLNMGILVSPLLA  117

P31442.2       69   RVGRRPVILVGMSIFMLATLVAVTTSSLTVLIAASAMQGMGTGVGGVMARTLPRDLYERTQLRHANSLLNMGILVSPLLA  148

P37597.3       67   RYGRKPVLLIGLTIFALGSLGMLWVENAATLLVLRFVQAVGVCAAAVIWQALVTDYYPSQKVNRIFAAIMPLVGLSPALA  146

B7NF27.1       64   RSGRKPVAIPGAALFIIASVFCSLAETSTLFLAGRFLQGLGAGCCYVVAFAILRDTLDDRRRAKVLSLLNGITCIIPVLA  143

B1LL36.1       64   RSGRKPVAIPGAALFIIASVFCSLAETSTLFLAGRFLQGLGAGCCYVVAFAILRDTLDDRRRAKVLSLLNGITCIIPVLA  143

B7NR12.1       64   RSGRKPVAIPGAALFIIASVFCSLAETSTLFLAGRFLQGLGAGCCYVVAFAILRDTLDDRRRAKVLSLLNGITCIIPVLA  143

A7ZTR5.1       64   RSGRKPVAIPGAALFIIASVFCSLAETSTLFLAGRFLQGLGAGCCYVVAFAILRDTLDDRRRAKVLSLLNGITCIIPVLA  143

B1X9T9.1       64   RSGRKPVAIPGAALFIIASVFCSLAETSTLFLAGRFLQGLGAGCCYVVAFAILRDTLDDRRRAKVLSLLNGITCIIPVLA  143

A8A6H2.1       64   RSGRKPVAIPGAALFIIASVFCSLAETSTLFLAGRFLQGLGAGCCYVVAFAILRDTLDDRRRAKVLSLLNGITCIIPVLA  143

P28246.4       71   SFGRKPVVLGGTLVFAAAAVACALANTIDQLIVMRFFHGLAAAAASVVINALMRDIYPKEEFSRMMSFVMLVTTIAPLMA  150

Q0TAZ8.1       64   RSGRKPVAIPGAALFIIASVFCSLAETSALFLAGRFLQGLGAGCCYVVAFAILRDTLDDRRRAKVLSLLNGITCIIPVLA  143

B7UMH7.1       64   RSGRKPVAIPGAALFIIASVFCSLAETSALFLAGRFLQGLGAGCCYVVAFAILRDTLDDRRRAKVLSLLNGITCIIPVLA  143

B7N2F5.1       64   RSGRKPVAIPGAALFIIASVFCSLAETSALFLAGRFLQGLGAGCCYVVAFAILRDTLDDRRRAKVLSLLNGITCIIPVLA  143

A1AHP3.1       64   RSGRKPVAIPGAALFIIASVFCSLAETSALFLAGRFLQGLGAGCCYVVAFAILRDTLDDRRRAKVLSLLNGITCIIPVLA  143

B5YXB4.1       64   RSGRKPVAIPGAALFIITSVFCSLAETSTLFLAGRFLQGLGAGCCYVVAFAILRDTLDDRRRAKVLSLLNGITCIIPVLA  143

P39386.1       74   RIGRRPVLITGALIFTLACAATMFTTSMTQFLIARAIQGTSICFIATVGYVTVQEAFGQTKGIKLMAIITSIVLIAPIIG  153

Q8XB84.1       74   RIGRKPVLITGALIFTLACAATMFTTSMTQFLIARAIQGTSICFIATVGYVTVQEAFGQTKGIKLMAIITSIVLIAPIIG  153

P0AEY8.1       78   RIGRRPVMLAGVVWFIVTCLAILLAQNIEQFTLLRFLQGISLCFIGAVGYAAIQESFEEAVCIKITALMANVALIAPLLG  157

P31474.1       72   RFGTRRIFTLAVSLFTLGSLACALSNSLPQLVVFRVIQGIGGAMMMPVARLALLRAYPRNELLPVLNFVAMPGLVGPILG  151

P77726.2       74   RIGRKPLIVGGLAVFAAGSVIAALSDSIWGIILGRALQGSGAIAAAVM--ALLSDLTREQNRTKAMAFIGVSFGITFAIA  151

P43531.2       97   AIGRKPVMVTALLLASICTLLSTMMTSWHGILIMRALIGLSLSGVAAVGMTYLSEEIHPSFVAFSMGLYISGNSIGGMSG  176

P76269.2       77   MFGYRRIYKCGLVVFLLSSLFCALSDSLQMLTLARVIQGFGGAALMSVNTALIRLIYPQRFLGRGMGINSFIVAVSSAAG  156

P0AEJ0.1       75   RVGEVKLFLWSTIAFAIASWACGVSSSLNMLIFFRVIQGIVAGPLIPLSQSLLLNNYPPAKRSIALALWSMTVIVAPICG  154

P02981.1       69   RFGRRPVLLASLLGATIDYAIMATTPVLWILYAGRIVAGI-TGATGAVAGAYIADITDGEDRARHFGLMSACFGVGMVAG  147

P02982.2       69   RFGRRPVLLVSLAGAAVDYAIMATAPFLWVLYIGRIVAGI-TGATGAVAGAYIADITDGDERARHFGFMSACFGFGMVAG  147


Query_EmrD     118  PLIGGLLDTMWNWRACYLFLLALCAGVTFSMARWM--P   ET--RPV-----DAPRTRL[4]K    TLFGNSGF---N  179

P31442.2       149  PLIGGLLDTMWNWRACYLFLLVLCAGVTFSMARWM--P   ET--RPV-----DAPRTRL[4]K    TLFGNSGF---N  210

P37597.3       147  PLLGSWLLVHFSWQAIFATLFAITVVLILPIFWLK--P   TT--KAR-----NNSQDGL[2]T    DLLRSKTY---R  206

B7NF27.1       144  PVLGHLIMLKFPWQSLFWTMATMGIAVLMLSLFIL--K   ET--RPAAPTNSDKPREN-   S    ESLLNRFF---L  205

B1LL36.1       144  PVLGHLIMLKFPWQSLFWTMATMGIAVLMLSLFIL--K   ET--RPAAPAASDKPREN-   S    ESLLNRFF---L  205

B7NR12.1       144  PVLGHLIMLKFPWQSLFWTMATMGIAILMLSLFIL--K   ET--RPAAPAASDKPREN-   S    ESLLNRFF---L  205

A7ZTR5.1       144  PVLGHLIMLKFPWQSLFWTMAMMGIAVLMLSLFIL--K   ET--RPASPAASDKPREN-   S    ESLLNRFF---L  205

B1X9T9.1       144  PVLGHLIMLKFPWQSLFWAMAMMGIAVLMLSLFIL--K   ET--RPAAPAASDKPREN-   S    ESLLNRFF---L  205

A8A6H2.1       144  PVLGHLIMLKFPWQSLFWAMAMMGIAVLMLSLFIL--K   ET--RPASPAASDKPREN-   S    ESLLNRFF---L  205

P28246.4       151  PIVGGWVLVWLSWHYIFWILALAAILASAMIFFLI--K   ET--LPPERRQPFHIRTTI[3]A    ALFRHKRV---L  216

Q0TAZ8.1       144  PVLGHLIMLKFPWQSLFWTMATMGIAVLMLSLFIL--K   ET--RPAAPAASDKPREN-   S    ESLLNRFF---L  205

B7UMH7.1       144  PVLGHLIMLKFPWQSLFWTMATMGIAVLMLSLFIL--K   ET--RPAAPTTSDKPREN-   S    ESLLNRFF---L  205

B7N2F5.1       144  PVLGHLIMLNFPWQSLFWTMAIMGVAVLMLSLFIL--K   ET--RPAAPAASDKPREN-   S    ESLLNRFF---L  205

A1AHP3.1       144  PVLGHLIMLKFPWQSLFWTMATMGIALLMLSLFIL--K   ET--RPAAPTTSDKPREN-   S    ESLLNRFF---L  205

B5YXB4.1       144  PVLGHLIMLKFPWQSLFWTMAIMGIAVLMLSLFIL--K   ET--RPAAPAASDKSREN-   S    ESLLNRFF---L  205

P39386.1       154  PLSGAALMHFMHWKVLFAIIAVMGFISFVGLLLAM--P   ETvkRGAVPFSAKSVLRDF   R    NVFCNRLF---L  218

Q8XB84.1       154  PLSGAALMHFVHWKVLFAIIAVMGFISFVGLLLAM--P   ETvkRGAVPFSAKSVLRDF   R    NVFCNRLF---L  218

P0AEY8.1       158  PLVGAAWIHVLPWEGMFVLFAALAAISFFGLQRAM--P   ET----ATRIGEKLSLKEL[4]K    LVLKNGRF---V  222

P31474.1       152  PVLGGVLVTWATWHWIFLINIPIGIAGLLYARKHM--P   NFt---TARRRFDITGFLL[4]L[48]DLFKTRTF---S  265

P77726.2       152  MVLGPIITHKLGLHALFWMIAILATTGIALTIWVV--P[8]RE---------SGMVKGSF[4]A[ 2]RLLKLNFG----  220

P43531.2       177  RLISGVFTDFFNWRIALAAIGCFALASALMFWKIL--P   ESr---HFRPTSLRPKTLF[3]R    LHWRDRGLpllF  244

P76269.2       157  PTIAAAILSIASWKWLFLINVPLGIIALLLAMRFL--P   PNg------SRASKPRFDL[4]M[52]DLLRIPLF---S  271

P0AEJ0.1       155  PILGGYISDNYHWGWIFFINVPIGVAVVLMTLQTLrgR   ETr---TERRRIDAVGLAL[4]I[49]SLFKSRNF---T  271

P02981.1       148  PVAGGLLG-AISLHAPFLAAAVLNGLNLLLGCFLM--Q   ESh----KGERRPMPLRAF[4]S    FRWARGMT---I  211

P02982.2       148  PVLGGLMG-GFSPHAPFFAAAALNGLNFLTGCFLL--P   ESh----KGERRPLRREAL[4]S    FRWARGMT---V  211


Query_EmrD     180  CYLLMLIGGLAGIAAFEACSGVLMG--AVLGLSSMTVSILFILPIPA-AFFGAWFAGRP----NK-RFSTLMWQSVICCL  251

P31442.2       211  CYLLMLIGGLAGIAAFEACSGVLMG--AVLGLSSMTVSILFILPIPA-AFFGAWFAGRP----NK-RFSTLMWQSVICCL  282

P37597.3       207  GNVLIYAACSASFFAWLTGSPFIL---SEMGYSPAVIGLSYVPQTIA-FLIGGYGCRAA---LQK-WQGKQLLPWLLVLF  278

B7NF27.1       206  SRVVITTLSVSVILTFVNTSPVLLM--EIMGFERGEYATIMALTAGV-SMTVSFSTPFA---LGI-FKPRTLMITSQVLF  278

B1LL36.1       206  SRVVITTLSVSVILTFVNTSPVLLM--EIMGFERGEYATIMALTAGV-SMTVSFSTPFA---LGI-FKPRTLMITSQVLF  278

B7NR12.1       206  SRVVITTLSVSVILTFVNTSPVLLM--EIMGFERGEYATIIALTAGV-SMTVSFSTPFA---LGI-FKPRTLMITSQVLF  278

A7ZTR5.1       206  SRVVITTLSVSVILTFVNTSPVLLM--EIMGFERGEYATIMALTAGV-SMTVSFSTPFA---LGI-FKPRTLMITSQVLF  278

B1X9T9.1       206  SRVVITTLSVSVILTFVNTSPVLLM--EIMGFERGEYATIMALTAGV-SMTVSFSTPFA---LGI-FKPRTLMITSQVLF  278

A8A6H2.1       206  SRVVITTLSVSVILTFVNTSPVLLM--EIMGFERGEYATIMALTAGV-SMTVSFSTPFA---LGI-FKPRTLMITSQVLF  278

P28246.4       217  SYMLASGFSFAGMFSFLSAGPFVYI--EINHVAPENFGYYFALNIVF-LFVMTIFNSRF---VRR-IGALNMFRSGLWIQ  289

Q0TAZ8.1       206  SRVVITTLSVSVILTFVNTSPVLLM--EIMGFERGEYATIMALTAGV-SMTVSFSTPFA---LGI-FKPRTLMITSQVLF  278

B7UMH7.1       206  SRVVITTLSVSVILTFVNTSPVLLM--EIMGFERGEYATIMALTAGV-SMTVSFSTPFA---LGI-FKPRTLMITSQVLF  278

B7N2F5.1       206  SRVVITTLSVSVILTFVNTSPVLLM--EIMGFERGEYATIMALTAGV-SMTVSFSTPFA---LGI-FKPRTLMITSQVLF  278

A1AHP3.1       206  SRVVITTLSVSVILTFVNTSPVLLM--EIMGFERGEYATIMALTAGV-SMTVSFSTPFA---LGI-FKPRTLMITSQVLF  278

B5YXB4.1       206  SRVVITTLSVSVILTFVNTSPVLLM--EIMGFERGEYATIMALTAGV-SMTVSFSTPFA---LGI-FKPRTLMITSQVLF  278

P39386.1       219  FGAATISLSYIPMMSWVAVSPVILI--DAGSLTTSQFAWTQVPVFGA-VIVANAIVARF---V-KdPTEPRFIWRAVPIQ  291

Q8XB84.1       219  FGAATISLSYIPMMSWVAVSPVILI--DAGGLTTSQFAWTQVPVFGA-VIVANAIVARF---V-KdPTEPRFIWRAVPIQ  291

P0AEY8.1       223  AGALALGFVSLPLLAWIAQSPIIII--TGEQLSSYEYGLLQVPIFGA-LIAGNLLLARL---TSR-RTVRSLIIMGGWPI  295

P31474.1       266  IGIVGNIATRLGTGCVPFLMPLMLQ--VGFGYQAFIAGCMMAPTALG-SIIAKSMVTQV---LRR-LGYRHTLVGITVII  338

P77726.2       221  ----IMCLHILLMSTFVALPGQLADa----GFPAAEHWKVYLATMLIaFGSVVPFIIYA----EVkRKMKQVFVFCVGLI  288

P43531.2       245  AEGFLLMGSFVTLFNYIGYRLMLSP----WHVSQAVVGLLSLAYLTG-----TWSSPKAgtmTTR-YGRGPVMLFSTGVM  314

P76269.2       272  LSICTSVCSFCAQMLAMVSLPFYLQ--TVLGRSEVETGLLLTPWPLA-TMVMAPLAGYL---IER-VHAGLLGALGLFIM  344

P0AEJ0.1       272  IGCLCISLAYMLYFGAIVLLPQLLQ--EVYGYTATWAGLASAPVGII-PVILSPIIGRF---AHK-LDMRRLVTFSFIMY  344

P02981.1       212  VAALMTVFFIMQLVGQVPAALWVIFgeDRFRWSATMIGLSLAVFGILhALAQAFVTGPA---TKR-FGEKQAIIAGMAAD  287

P02982.2       212  VAALMAVFFIMQLVGQVPAALWVIFgeDRFHWDATTIGISLAAFGILhSLAQAMITGPV---AAR-LGERRALMLGMIAD  287


Query_EmrD     252  LAGLLM-WIPDW[4]NVWTLLIPAALFFFGAGMLFPLATSGAMEPFPF-LAGTAGALVGGLQNI------------   -  314

P31442.2       283  LAGLLM-WIPDW[4]NVWTLLVPAALFFFGAGMLFPLATSGAMEPFPF-LAGTAGALVGGLQNIGSGVLASLSAML   P  358

P37597.3       279  AVSVIAtWAAGF[4]SLVEILIPFCVMAIANGAIYPIVVAQALRPFPH-ATGRAAALQNTLQLGLCFLASLVVSWL   I  355

B7NF27.1       279  LAAGIT-LAVSP   SHAVSLFGITLICAGFSVGFGVAMSQALGPFSL-RAGVASSTLGIAQVCGSSLWIWLAAVV   G  350

B1LL36.1       279  LAAGIT-LAVSP   SHAVSLFGITLICAGFSVGFGVAMSQALGPFSL-RAGVASSTLGIAQVCGSSLWIWLAAVV   G  350

B7NR12.1       279  LAAGIT-LAVSP   SHAVSLFGITLICAGFSVGFGVAMSQALGPFSL-RAGVASSTLGIAQVCGSSLWIWLAAVV   G  350

A7ZTR5.1       279  LAAGIT-LAVSP   SHAVSLFGITLICAGFSVGFGVAMSQALGPFSL-RAGVASSTLGIAQVCGSSLWIWLAAVV   G  350

B1X9T9.1       279  LAAGIT-LAVSP   SHAVSLFGITLICAGFSVGFGVAMSQALGPFSL-RAGVASSTLGIAQVCGSSLWIWLAAVV   G  350

A8A6H2.1       279  LAAGIT-LAVSP   SHAVSLFGITLICAGFSVGFGVAMSQALGPFSL-RAGVASSTLGIAQVCGSSLWIWLAAVV   G  350

P28246.4       290  FIMAAW-MVISA[4]GFWSLVVGVAAFVGCVSMVSSNAMAVILDEFPH-MAGTASSLAGTFRFGIGAIVGALLSLA   T  365

Q0TAZ8.1       279  LAAGIT-LAVSP   SHAVSLFGITLICAGFSVGFGVAMSQALGPFSL-RAGVASSTLGIAQVCGSSLWIWLAAVV   G  350

B7UMH7.1       279  LAAGIT-LAVSP   SHAVSLFGITLICAGFSVGFGVAMSQALGPFSL-RAGVASSTLGIAQVCGSSLWIWLAAVV   G  350

B7N2F5.1       279  LAAGIT-LAVSP   SHAVSLFGITLICAGFSVGFGVAMSQALGPFSL-RAGVASSTLGIAQVCGSSLWIWLAAVV   G  350

A1AHP3.1       279  LAAGIT-LAVSP   SHAVSLFGITLICAGFSVGFGVAMSQALGPFSL-RAGVASSTLGIAQVCGSSLWIWLAAVV   G  350

B5YXB4.1       279  LAAGIT-LTVSP   SHAVSLFGITLICAGFSVGFGVAMSQALGPFSL-RAGVASSTLGIAQVCGSSLWIWLAAVV   G  350

P39386.1       292  LVGLSLlIVGNL[4]VWLWSVLGTSLYAFGIGLIFPTLFRFTLFSNKL-PKGTVSASLNMVILMVMSVSVEIGRWL   W  368

Q8XB84.1       292  LVGLALlIIGNL[4]VWLWSVLGTSLYAFGIGLIFPTLFRFTLFSNNL-PKGTVSASLNMVILMVMSVSVEIGRWL   W  368

P0AEY8.1       296  MIGLLVaAAATV[4]AYLWMTAGLSIYAFGIGLANAGLVRLTLFASDM-SKGTVSAAMGMLQMLIFTVGIEISKHA[1]-  372

P31474.1       339  GLMIAQfSLQSP[2]AIWMLILPLFILGMAMSTQFTAMNTITLADLTDdNASSGNSVLAVTQQLSISLGVAVSAAV[5]G  419

P77726.2       289  VVAEIVlWNAQT   QFWQLVVGVQLFFVAFNLMEALLPSLISKESPAgYKGTAMGVYSTSQFLGVAIGGSLGGWI[1]G  363

P43531.2       315  LFGLLMtLFSSL   --WLIFAGMLLFSAGFFAAHSVASSWIGPRAKR-AKGQASSLYLFSYYLGSSIAGTLGGVF[2]-  386

P76269.2       345  AAGLFS-LVLLP[3]ADINIIWPMILCGAGFGLFQSPNNHTIITSAPReRSGGASGMLGTARLLGQSSGAALVALM[4]G  424

P0AEJ0.1       345  AVCFYW-RAYTF[4]DFGASAWPQFIQGFAVACFFMPLTTITLSGLPPeRLAAASSLSNFTRTLAGSIGTSITTTM[5]S  426

P02981.1       288  ALGYVL-LAFAT[2]---WMAFPIMILLASGGIGMPALQAMLSRQVDDdHQGQLQGSLAALTSLTSITGPLIVTAI   Y  359

P02982.2       288  GTGYIL-LAFAT[2]---WMAFPIMVLLASGGIGMPALQAMLSRQVDEeRQGQLQGSLAALTSLTSIVGPLLFTAI   Y  359


Query_EmrD          -----    ---------------------   --   ------------    ------      

P31442.2       359  QT-GQ    GSLGLLMTLMGLLIVLCWLPL   AT[4]QG---QPV----    ------      394

P37597.3       356  SI-ST    PLLTTTSVMLSTVVLVALGYM   MQ[4]VG---CQNHGNA    EVAHSE[ 2]  403

B7NF27.1       351  I--GA    WNMLIGILIACSIVSLLLIMF   VA   PG---RPVAAHE    EIHHHA      391

B1LL36.1       351  I--GA    WNMLIGILIACSIVSLLLIMF   VA   PG---RPVAAHE    EIHHHA      391

B7NR12.1       351  I--GA    WNMLIGILIACSIVSLLLIMF   IA   PG---RPVAAHE    EIHHHA      391

A7ZTR5.1       351  I--GA    WNMLIGILIACSIVSLLLIMF   VA   PG---RPVAAHE    EIHHHA      391

B1X9T9.1       351  I--GA    WNMLIGILIACSIVSLLLIMF   VA   PG---RPVAAHE    EIHHHA      391

A8A6H2.1       351  I--GA    WNMLIGILIACSIVSLLLIMF   VA   PG---RPVAAHE    EIHHHA      391

P28246.4       366  FN-SA    WPMIWSIAFCATSSILFCLYA   SR   PK---KR-----    ------      396

Q0TAZ8.1       351  I--GA    WNMLIGILIACSIVSLLLIMF   VA   PG---RPVAAHE    EIHHHA      391

B7UMH7.1       351  I--GA    WNMLIGILIACSIVSLLLIMF   VA   PG---RPVAAHE    EIHHHA      391

B7N2F5.1       351  I--GA    WNMLIGILIACSIVSLLLIMF   VA   PG---RPVAAHE    EIHHHA      391

A1AHP3.1       351  I--GA    WNMLIGILIACSIVSLLLIMF   VA   PG---RPVAAHE    EIHHHA      391

B5YXB4.1       351  I--SA    WNMLIGILIACSIVSLLLIMF   VA   PG---RPVTAHE    EIHHHA      391

P39386.1       369  FNgGR    LPFHLLAVVAGVIVVFTLAGL   LN[1]-------VRQHQ    AAELVE[ 2]  410

Q8XB84.1       369  FNgGR    LPFHLLAVVAGVIVVFTLAGL   LN[1]-------VRQHQ    AAELAE[ 2]  410

P0AEY8.1       373  LNgGN    GLFNLFNLVNGILWLSLMVIF   -L[4]MGn------SHE[ 1]------      410

P31474.1       420  MEgTT[ 3]-QFHYTFITMGIITVASAAMF   ML[4]DGnnl--IKRQR[ 5]RVPSES[ 1]  475

P77726.2       364  MFdGQ    GVFLAGAMLAAVWLTVASTMK[6]SL[4]PAn----IAANE[17]AEEEHS[22]  454

P43531.2       387  -NyGW    NGVGAFIALMLVIALLVGTRL   HR[3]-A----------    ------      417

P76269.2       425  DNgTH[ 1]SLMAAAILAVIAACVSGLRIT[3]-S[2]------------    ------      457

P0AEJ0.1       427  MHhAQ[11]AQAMYSQLEGLGMTQQQASGW   IA[4]NQgli--ISANE[17]----FA[14]  512

P02981.1       360  AAsAS    TWNGLAWIVGAALYLVCLPAL   RR[1]---------AWS    RA---T[ 2]  396

P02982.2       360  AAsIT    TWNGWAWIAGAALYLLCLPAL   RR[1]---------LWS    GAGQRA[ 2]  399
