# Supplementary material for: Molecular diversity and structural profiling of the EmrD efflux pump in multidrug-resistant uropathogenic Escherichia coli
Source: PLoS One. 2026 Nov 11;21(11):e0362259. doi: 10.1371/journal.pone.0362259 (PMC13754136; doi:10.1371/journal.pone.0362259)
Supplement: Supplementary file 3 [file pone.0362259.s005.docx]

**Date:** July 15, 2026

**To:** Dr. Daniel F. M. Monte, Ph.D.

Academic Editor, *PLOS ONE*

**Dear Dr. Monte and the Editorial Board,**

Thank you very much for providing us with the opportunity to revise our manuscript, titled **"Molecular diversity and structural profiling of the EmrD efflux pump in multidrug-resistant uropathogenic Escherichia coli"** (Manuscript ID: **PONE-D-26-16505**), for publication in *PLOS ONE*. We are deeply grateful to you and the peer reviewers for the constructive feedback, encouraging assessments, and highly precise technical suggestions regarding our work.

We have taken all comments into serious consideration and have thoroughly revised the manuscript to address every point raised. Specifically, we have significantly moderated our tone throughout the text—shifting our conclusions to hypotheses—to reflect the exploratory, pilot nature of this regional molecular diversity survey. Furthermore, we have corrected all data discrepancies, adjusted our figure and table formatting, and updated our methodology text to fully satisfy the journal’s scientific criteria. **Additionally, in accordance with the journal's data integrity requirements, we have compiled and uploaded the original, uncropped raw gel electrophoresis images as a supporting file ("S1_Raw_Images").**

In the pages that follow, we provide a comprehensive, point-by-point response detailing how and where each comment has been addressed. The specific line numbers referenced in our responses correspond directly to the clean version of our revised manuscript (Manuscript.docx). For your convenience, all alterations have also been tracked visually within our accompanying document, Revised Manuscript with Track Changes.docx.

Thank you once again for your time, guidance, and support of our research. We hope that these extensive revisions render our manuscript fully acceptable for publication in *PLOS ONE*.

**Dr. Iman H. F. Alnaqshabandy**

Head of the Scientific Research Center

University of Duhok, Duhok, Kurdistan Region, Iraq

Email: iman.fadhiladeen@uod.ac

## **Point-by-Point Responses to Editorial and Reviewer Comments**

### **I. Responses to Editorial Comments**

#### **Editor Comment 1: Style Requirements**

*Please ensure that your manuscript meets PLOS ONE's style requirements, including those for file naming, heading styles, and author affiliation formatting.*

**Response:** Thank you for pointing out these formatting requirements. We have thoroughly revised the manuscript layout to strictly adhere to the *PLOS ONE* style guidelines. Specifically, we have implemented the following changes:

- **Author Affiliations:** Consolidated the duplicate entries into a single unified affiliation block to remove redundancy, formatting it from smallest to largest component without forbidden abbreviations.
- **Corresponding Author:** Updated the corresponding author block to include the author's initials in parentheses directly after the email address.
- Section Headings: Removed all numerical prefixes (e.g., changing "2.1.", "2.2.", "3.1.") from all Level 1 and Level 2 headings throughout the document.
- **Font Hierarchy:** Standardized the text structure using Times New Roman, setting the main body text to 11pt (double-spaced) and establishing a clear hierarchy with 18pt bold font for Level 1 headings and 16pt bold font for Level 2 headings.
- **Figure Citations:** Corrected all in-text citations of figures to follow the exact journal style (e.g., "Fig 1" and "Fig 2" instead of "Fig. 1" or "Fig. 2").

#### **Editor Comment 2: Data Availability Statement**

*Please note that your Data Availability Statement is currently missing the accession number of each dataset can’t access... We therefore suggest that you provide this information now...*

**Response:** Thank you for highlighting this requirement. All nucleotide sequences generated for the *emrD* gene in this study have been deposited in the NCBI GenBank database and are fully public. They can be accessed using the accession numbers **PZ196343 through PZ196352**. This information is explicitly stated in the Materials and methods section (Lines 153-154) and the Data Availability statement (Lines 403-405) of our revised manuscript.

#### **Editor Comment 3: Original Blot/Gel Images**

*PLOS ONE now requires that authors provide the original uncropped and unadjusted images underlying all blot or gel results reported... In your cover letter, please note whether your blot/gel image data are in Supporting Information or posted at a public data repository...*

**Response:** We fully comply with *PLOS ONE*'s data integrity guidelines. We have compiled the original, uncropped, and unaltered raw gel electrophoresis images for our PCR assays (corresponding to the data shown in Fig 1). These raw files have been uploaded alongside our revision as a single supporting file titled **"S1_Raw_Images"**. We have also explicitly stated this in our updated Cover Letter as requested.

#### **Editor Comment 4: Recommended Citations**

*If the reviewer comments include a recommendation to cite specific previously published works, please review and evaluate these publications...*

**Response:** We have carefully reviewed all comments from the Academic Editor and reviewers. No specific recommendations or demands to cite external publications were made. Where reviewers suggested contextual improvements to the manuscript, we have updated our text accordingly using relevant scientific literature.

### **Additional Editor Comments: Scope and Over-Extrapolation**

*The main concern is that several conclusions extend beyond the evidence presented... no functional experiments were performed... interpretations suggesting adaptive evolution, altered transporter function, or direct contributions to multidrug resistance should be significantly moderated and presented as hypotheses rather than conclusions... limitations of the study should be more explicitly acknowledged...*

**Response:**

We sincerely thank the Editor for this invaluable guidance. We fully agree that in the absence of *in vitro* functional validation, our structural and phylogenetic findings are best positioned as exploratory and hypothesis-generating. We have thoroughly revised the manuscript to address these concerns:

1. **Moderated Tone in the Abstract:** We have systematically toned down our interpretations in the Abstract. Definitive terms like *"evolutionary heterogeneity"* have been replaced with *"local genetic diversity"*, and we have explicitly reframed our structural conclusions to suggest that these genomic variations *"may potentially influence"* efflux efficiency, emphasizing the need for future functional validation.
2. **Reframed Conclusion & the "Induced-Fit" Hypothesis:** In the Conclusion, we have carefully moderated our claims while preserving our core theoretical insights. We have explicitly reframed our "induced-fit" evolutionary model as a **proposed hypothesis** regarding how MFS transporters adapt, rather than a proven mechanism, acknowledging that functional assays are strictly required to confirm this model.
3. **Dedicated "Study Limitations" Section:** As requested, we have included an explicit "Study Limitations" subsection at the end of the Discussion. This section explicitly notes the pilot nature of the study, the small sample size (*n = 10*), and the lack of molecular dynamics simulations, RT-qPCR transcript quantification, or site-directed mutagenesis to kinetically validate the *V170A/V304I* variants. We explicitly state that future functional studies are required to definitively correlate these genotypes with altered minimum inhibitory concentrations (MICs).
4. **Tempered Evolutionary Claims:** We have systematically revised our evolutionary interpretations throughout the Discussion to avoid unsupported assertions regarding active selection pressures or dissemination pathways, presenting these instead as exploratory genomic characterizations.

**II. Responses to Reviewer #1**

#### **Comment 1: Over-Extrapolation & Lack of Functional Assays**

#### *The study demonstrates only the universal presence of emrD, the existence of sequence variation, and the structural localisation of these substitutions within relevant transmembrane regions. No functional experiments were conducted to demonstrate altered efflux activity, changes in substrate specificity, differences in MIC values, altered gene expression, or any phenotypic consequence associated with the identified variants.*

#### **Response:** We completely agree with the reviewer’s excellent critique. We acknowledge that without direct functional, kinetic, or expression assays, implying a definitive causal link between the identified variants and a resistance phenotype is speculative.

#### Accordingly, we have comprehensively revised the text to ensure a strict distinction between empirical observations and speculative interpretations. All assertive, causal verbs have been changed to cautious, tentative language. Furthermore, we have added a dedicated **Study Limitations** subsection to the Discussion explicitly acknowledging the absence of functional validation (e.g., RT-qPCR, MIC comparisons of isolated mutants, and efflux assays) and reframing the work as an exploratory, hypothesis-generating molecular survey.

#### **Location of Changes in the Revised Manuscript:**

#### *Results (Amino acid substitutions and structural localization in EmrD, Lines 254–256):* "...suggests localized sequence polymorphism at this residue. However, given its conservative biochemical nature, further functional and structural investigations are required to determine if this variation holds any structural or phenotypic significance, or simply reflects neutral evolutionary divergence."

#### *Results (Amino acid substitutions and structural localization in EmrD, Lines 274–276):* "This specific mutational profile requires further structural and functional characterization to determine its effects on protein conformation and substrate transport."

#### *Discussion (Physiological role: virulence and host-pathogen interaction, Lines 293–296):* "Howevers, because *emrD* is a conserved core gene found in both pathogenic and non-pathogenic E. coli, its universal presence likely reflects genomic conservation rather than a specific role in UPEC virulence or host colonization."

#### *Discussion (Co-resistance and biofilm potential, Lines 302–304):* "Such co-selection suggests that emrD variants are maintained alongside β-lactamase genes in Iraqi UPEC isolates. Previous studies have linked efflux pump regulation with biofilm formation and motility."

#### **Comment 2: Evolutionary Interpretations & Genetic Drift**

#### *The manuscript repeatedly describes rare variants as “specialised adaptations”... yet no robust evolutionary analyses were performed to support such claims. The observed substitutions may simply reflect neutral drift...*

#### **Response:**

#### This is an insightful point. We agree that sequence rarity alone does not equal adaptive selection and that in the absence of *dN/dS* ratios, codon-level selection tests, or large-scale population genomics, these variants may simply reflect neutral genetic drift or lineage-specific background variation. We have removed all claims characterizing these mutations as "specialized adaptations" or "structural fine-tuning."

#### **Location of Changes in the Revised Manuscript:**

#### *Discussion (Phylogenetic diversity and evolutionary dynamics, Lines 321–324):* "Importantly, the evolutionary significance of variants such as V304I remains uncertain. Without *dN/dS* analysis or population-scale genomic data, these substitutions are more likely to reflect neutral genetic drift or lineage-specific polymorphism than adaptive evolution."

#### **Comment 3: Interpretation of Positional Structural Mapping**

*The structural discussion is conceptually interesting... Nevertheless, the manuscript repeatedly exceeds the interpretative limits permitted by positional structural mapping alone... despite the absence of molecular dynamics simulations, docking analyses, stability calculations, or any direct structural modelling beyond residue localisation.*

**Response:** We appreciate this valuable guidance. We have carefully modified our structural discussions to ensure that our structural insights are framed strictly as positional hypotheses rather than definitive biochemical consequences. While we have retained the conceptual literature context regarding the MFS rocker-switch mechanism, we have explicitly clarified the limits of our static mapping model.

- **Location of Changes in the Revised Manuscript:**
  - *Discussion (Structural implications of the emrD mutations, Lines 345–347, 351–353, 357–360, and 362–364):*
    "...highlights their limited distribution. However, their evolutionary significance cannot be determined from sequence data alone and requires further functional and population-level investigation.

This is consistent with the possibility that *emrD* variants may undergo structural diversification under antibiotic selection pressures, although this remains speculative.

Although these substitutions could theoretically influence the induced-fit mechanism described for MFS transporters, their effects on transporter dynamics and substrate recognition remain unknown without molecular dynamics simulations and functional validation.

Therefore, the structural and evolutionary significance of the Cluster II variants remains unresolved and should be regarded as a hypothesis requiring further structural and functional investigation."

#### **Comment 4: Technical Quality, Contrast, and Interpretability of Figures**

*The figures are broadly coherent with the manuscript text but present several limitations. Figure 1 suffers from poor visual quality and insufficient contrast. Figure 2 has limited resolution and poorly interpretable bootstrap support, whilst Figure 3, although conceptually the strongest, represents only positional mapping rather than rigorous structural analysis. The figure legends generally correspond well to the manuscript; however, in some cases they reinforce mechanistic interpretations that are not adequately supported by the data.*

**Response:** We thank the reviewer for this constructive feedback regarding our figures and legends. We have revised our visual presentations to adhere to strict high-resolution standards and have adjusted the captions to reflect a neutral, observational tone:

- **Figure 1:** In accordance with the editorial guidelines, this image has been updated to optimize contrast and background clarity.
- **Figure 2:** To ensure perfect clarity of the bootstrap values, this figure was handled natively as a vector-based text document before conversion to PDF. Because the lines and text are stored as mathematical vector paths rather than static pixels, the figure maintains crisp legibility and infinite resolution at any zoom level.
- **Figure 3:** We completely agree with the reviewer that this figure represents a static positional mapping of residues rather than a dynamic or functional structural simulation. To ensure this limitation is explicitly clear to the reader, we have updated the figure legend to clarify the observational nature of the model (Location: Results section: Line number 271–272).

### **III. Responses to Reviewer #2**

#### **Comment 1: Affiliation Mapping Consistency**

*Affiliation Mapping: Please clarify why the affiliations are indicated with two different numbers if they refer to the same institution. This should be consolidated to avoid confusion.*

**Response:** We thank the reviewer for catching this administrative error. The affiliations have been carefully reviewed and consolidated under a single institutional number to remove redundancy.

- **Location of Changes: Title and Affiliations page (Page 1).**

#### **Comment 2: Formatting & Typos on Lines 68, 73, and 74**

*Line 68: “membrane [15,16]”. Ensure there is a space before the brackets... Line 73: Extra letters “Uropathogenic”... Line 74: Grammar correction: Change to "...responsible for 75% of uncomplicated..."*

**Response:** We appreciate this precise proofreading. All three identified issues have been corrected in accordance with the reviewer's instructions and the journal's guidelines:

- A space has been added before the citation brackets at Line 70.
- The duplicate/stray word "Uropathogenic" has been deleted at Line 75.
- The phrase has been grammatically corrected to "...responsible for 75% of uncomplicated..." at Line 76.

#### **Comment 3: Microbiological Context of Cloxacillin Resistance**

*Line 103: Regarding the mention of cloxacillin: It is important to note that E. coli is inherently resistant to cloxacillin. Reporting this as an "acquired resistance" or a significant finding might be seen as a lack of microbiological context.*

**Response:** We completely agree with this critical microbiological distinction. *E. coli* is naturally and inherently resistant to cloxacillin due to poor outer membrane permeability and intrinsic chromosomal mechanisms; framing it alongside acquired multidrug resistance profiles was biochemically inaccurate. We have removed any narrative characterizing cloxacillin resistance as an acquired or novel finding and added explicit context regarding its intrinsic nature.

- **Location of Changes in the Revised Manuscript:**
  - *Materials and Methods (Bacterial strains and DNA samples, Lines 110–112):*
    "Phenotypic resistance to cloxacillin was also uniformly observed, which was contextualized strictly as an intrinsic, natural property of *Escherichia coli* due to poor outer membrane permeability rather than an acquired resistance trait."

#### **Comment 4: Primer Concentration and Reaction Volume**

*Line 114 (Primer Concentration): There is a potential discrepancy in the reaction volume. You mention using 2 µL of a 5 µM stock, which results in 10 pmol per reaction. Please verify if the intended amount was 5 pmol (absolute amount) or if the stock concentration was different.*

**Response:**

We thank the reviewer for their careful calculation of our reaction dynamics. To eliminate any ambiguity regarding stock solutions and pipetting volumes, we have revised the text to state the definitive, final concentration of the primers within the reaction mixture. The manuscript has been updated to clarify that each primer was present at a standard final concentration of 0.5 µM.

- **Location of Changes in the Revised Manuscript:**
  - *Materials and Methods (Molecular detection of β-Lactamase and the emrD gene, Line 126):*
    "PCR reactions were carried out in a 20 µL total volume containing 10 µL of 2× PCR Master Mix (Promega, South Korea), each primer at a final concentration of 0.5 µM, 2 µL of DNA template, and nuclease-free water to the final volume."

#### **Comment 5: Table 1 Validation and Main Text Reference Citation**

*Line 119 (Table 1 & References): It is highly recommended to merge the primer data into a single, cohesive table. Currently, blaNDM appears twice (positions 1 and 6). Reference [21] must be cited in the main text. Example: "The primers used for β-lactamase gene detection were obtained from previous studies [21] and are summarized in Table 1."*

**Response:** We thank the reviewer for their careful eye toward our document layout. We have audited Table 1 and confirmed that all target genes are already consolidated into this single, cohesive table with no duplicate entries; blaNDM is listed exclusively at the first position, and emrD occupies the final position.

Per your recommendation, we have explicitly integrated the citation sentence for Reference [21] into the main body text of the Materials and Methods section right after our introduction of the target genes to introduce the primer origins smoothly.

- **Location of Changes in the Revised Manuscript:**
  - *Materials and Methods (Molecular detection of β-Lactamase and the emrD gene, Lines 123–124):*
    "The primers used for β-lactamase gene detection were obtained from previous studies [21], while the emrD target primers were designed in this study; all sequences are natively summarized in Table 1."

#### **Comment 6: E. coli K-12 Reference Strain Specification**

*Line 135: Specify the exact strain of E. coli K-12 used (e.g., MG1655, W3110).*

**Response:** We thank the reviewer for pointing out this missing specification. The reference sequence utilized for comparative analysis was *Escherichia coli* K-12 substrain MG1655. The text has been amended to reflect this specific strain designation.

- **Location of Changes in the Revised Manuscript:**
  - *Materials and Methods (Sequence processing and alignment, Line 148):*
    "The ten generated consensus sequences along with the reference emrD sequence retrieved from GenBank using *Escherichia coli* K-12 substrain MG1655 were then subjected to multiple sequence alignment..."

#### **Comment 7: Acronym Definition: MAFFT**

*Line 136: Define the acronym upon first use: MAFFT (Multiple Alignment using Fast Fourier Transform).*

**Response:** We apologize for this omission and the typographical oversight. The acronym has been fully defined upon its first appearance in the manuscript text, and the spelling of "MAFFT" has been corrected.

- **Location of Changes in the Revised Manuscript:**
  - *Materials and Methods (Sequence processing and alignment, Line 149):*
    "...subjected to multiple sequence alignment (MSA) using the MAFFT (Multiple Alignment using Fast Fourier Transform) algorithm implemented in the Benchling server."

#### **Comment 8: NCBI Database Accession Numbers**

*Line 139: Accession Numbers: I was unable to locate the mentioned accession numbers in the NCBI database. These must be public and searchable before publication.*

**Response:** We thank the reviewer for verifying our sequence records. At the time of the initial manuscript submission, these sequences were still undergoing standard administrative processing by the NCBI GenBank team. We requested immediate online publication upon completion of processing, and we are pleased to report that the records are now fully processed, un-embargoed, public, and searchable worldwide.

The active GenBank accession numbers for our 10 clinical isolates are (**PZ196343, PZ196344, PZ196345, PZ196346, PZ196347, PZ196348, PZ196349, PZ196350, PZ196351, PZ196352**), which are also explicitly noted in the Data Availability Statement.

#### **Comment 9: Selection Criteria for Phylogenetic Reference Sequences**

*Line 151 (Reference Sequences): Please provide a rationale for selecting exactly 25 reference sequences. Were these selected to represent different phylogroups, or were they simply the top 25 hits from a BLAST search?*

**Response:** We thank the reviewer for requesting this clarification. The 25 reference sequences were selected based on strict sequence homology using the NCBI BLASTp tool against the non-redundant (nr) protein database. Specifically, we retrieved the top 25 *E. coli* sequences that demonstrated the highest percentage of identity and maximum query coverage to our reference. This allowed us to calculate mutation frequencies against the closest known evolutionary matches. The manuscript text has been updated to explicitly state these homology criteria.

- **Location of Changes in the Revised Manuscript:**
  - *Materials and Methods (In Silico conservation and frequency analysis, Lines 167–168):*
    "...The protein sequence of the *E. coli* K-12 EmrD reference was used as a query against the non-redundant protein sequences (nr) database. A dataset of the top 25 representative *E. coli* reference sequences displaying the highest percentage of identity and maximum query coverage was retrieved to calculate the frequency..."

#### **Comment 10: Sequential Table Numbering & Citations**

*Line 152 (Table Numbering): Table 3 is cited before Table 2. Tables must be numbered sequentially according to their appearance in the text.*

**Response:** We thank the reviewer for this constructive note on manuscript flow. To improve readability and generalize the statement in the introductory section, we have removed the premature citation of the substitution frequency table from this paragraph entirely. This resolves the sequencing conflict, and all remaining tables have been verified to appear in strict chronological, sequential order throughout the manuscript text.

- **Location of Changes in the Revised Manuscript:**
  - *Materials and Methods (In Silico conservation and frequency analysis, Line 169):*
    "...A dataset of the top 25 representative E. coli reference sequences displaying the highest percentage of identity and maximum query coverage was retrieved to calculate the frequency of specific amino acid substitutions. Multiple sequence alignments were visualized..."

#### **Comment 11: Capitalization Adjustment for MDR**

*Line 156: Suggested phrasing: "...exhibited a uniform multidrug-resistant (MDR) …"*

**Response:** We thank the reviewer for catching this stylistic formatting issue. We have corrected the capitalization in the manuscript text to ensure proper lowercase formatting for "multidrug-resistant" in the middle of the sentence.

- **Location of Changes in the Revised Manuscript:**
  - *Results (Antibiotic resistance profiles, Line 173):*
    "...The ten clinical isolates examined in this study exhibited a uniform multidrug-resistant (MDR) phenotype..."

#### **Comment 12: Clinical Relevance of Cloxacillin Resistance in *E. coli***

*Line 157: Mentioning "100% resistance" to cloxacillin is technically accurate but clinically irrelevant for E. coli. I suggest removing or de-emphasizing this.*

**Response:** We completely agree with the reviewer's excellent clinical assessment. Because *E. coli* possesses intrinsic resistance to cloxacillin, highlighting this data adds little clinical value to the profile of acquired resistance traits. Following your recommendation, we have completely deleted the mention of cloxacillin resistance from this section of the text to keep the results focused exclusively on clinically relevant, acquired resistance patterns.

- **Location of Changes in the Revised Manuscript:**
  - The explicit mention of cloxacillin has been removed from the text string at Line 174.

#### **Comment 13: PCR Product Size Discrepancy**

*Line 165 (PCR Product Size): In Table 1, the expected size is 1,076 bp. However, the text mentions approximately 1,200 bp. This discrepancy (over 100 bp) needs to be explained or corrected to ensure the integrity of the PCR results.*

**Response:** We thank the reviewer for their meticulous observation. The mention of "approximately 1,200 bp" in the original text was a rough visual estimate from the initial gel run. To ensure absolute accuracy and consistency with Table 1, we have corrected the manuscript text to reflect the exact theoretical and observed amplicon size of 1,076 bp.

- **Location of Changes in the Revised Manuscript:**
  - *Results (PCR amplification of the emrD gene, Line 184):*
    "The PCR amplification of the emrD gene was successful for all 10 clinical E. coli isolates (DUH 01, DUH 02, DUH 03, DUH 04, DUH 05, DUH 06, DUH 09, DUH 10, DUH 11, and DUH 21). Agarose gel electrophoresis revealed clear, single DNA bands of the expected size, 1,076 bp (corresponding to the full-length coding sequence), for all samples..."

#### **Comment 14: Character Rendering / PDF Glitch**

*Line 200 (Character Rendering): The symbol “ ” (Beta) appears as a “Ghost Character” in my PDF reader. Please check if this is a rendering error or if there is actually a problem.*

**Response:** We thank the reviewer for bringing this rendering issue to our attention. This was indeed a PDF-conversion font-embedding error where the Greek symbol "β" (Beta) was not rendered correctly by the PDF engine, resulting in a blank square/ghost character.

We have converted all instances of the word "Beta" to the standardized, universally compatible Unicode symbol "β" and configured our PDF generation settings to embed all symbolic fonts. We have verified that the character now renders flawlessly as "β" in the revised manuscript PDF*.*

- **Location of Changes in the Revised Manuscript:**
  - Throughout the manuscript, specifically at Line 227 and surrounding text.

#### **Comment 15: Strain Nomenclature Standardization**

### **Line 204 (Strain Nomenclature): Standardize the strain IDs throughout the manuscript. The text alternates between space and hyphen (e.g., "DUH 04" vs. "DUH-04").**

### **Response:** We thank the reviewer for pointing out this inconsistency. We have thoroughly reviewed the entire manuscript, including all figures, tables, and supplementary materials, to standardize our strain nomenclature. All isolates are now consistently formatted using a space with a leading zero for single digits (e.g., "DUH 04", "DUH 10").

### **Location of Changes in the Revised Manuscript:** Corrected systematically throughout the entire text, tables, and figure labels of the manuscript.

#### **Comment 16: Phylogenetic Analysis Consistency**

*Line 205 (Phylogeny): There is a contradiction regarding the "basal" strain. Earlier, DUH 11 was identified as the divergent/basal outlier, but here DUH 01 is mentioned.*

**Response:** We thank the reviewer for this incredibly sharp observation. The reviewer is completely correct. **DUH 11** is indeed the true divergent, basal outlier of our dataset (and is *bla*SHV-positive), while **DUH 01** belongs to Cluster III (and is *bla*SHV-negative). Labeling DUH 01 as the "basal strain" in that sentence was an error.

We have revised the text to correctly identify DUH 01 by its clade (Cluster III) and reserved the "basal outlier" designation strictly for DUH 11.

- **Location of Changes in the Revised Manuscript:**
  - *Results (Distribution of β-lactamase resistance genes, Line 232):*
    "Notably, both strains in the intermediate phylogenetic clade (DUH 04 and DUH 10) and the Cluster III strain DUH 01 were negative for blaSHV. In contrast, the blaSHV gene was present in the majority of the reference-associated cluster (DUH 02, DUH 05, DUH 06) and the divergent, basal outlier DUH 11."

#### **Comment 17: Zotero Citations and Reference Standardization**

*Line 341 (Zotero/Citations): Before submission, ensure you "Unlink Citations" if using Zotero. Also, standardize journal names: either use full names or standard ISO abbreviations consistently for all entries.*

**Response:** We thank the reviewer for this helpful reminder regarding manuscript preparation.

1. **Unlinking Citations:** We have preserved an active, linked version of our manuscript for our records and successfully utilized Zotero’s "Unlink Citations" tool on the final submission draft. All reference fields have been flattened into plain, static text to prevent any software rendering issues during the journal’s typesetting process.
2. **Journal Standardization:** We have thoroughly audited the entire reference list. All journal names have been standardized to their official **standard ISO abbreviations with periods** (e.g., *Front. Microbiol.*, *Nat. Rev. Microbiol.*) to ensure perfect consistency across all bibliography entries.
3. **Correction of Entry Duplications:** We have removed the repeated title in Reference 7 (Line 419) and eliminated duplicate reference entries (such as Reference 26, which duplicated Reference 16) to ensure the final list is fully polished.

- **Location of Changes:** Throughout the entire **References** section (Lines 406 onward).

### **Comment 18: Repeated Title in References**

*Line 354: There is a repeated title in section 7. Please remove the duplication.*

**Response:** We thank the reviewer for identifying this duplication. We have removed the repeated title in Section 7 (Reference 7) to ensure the entry is clean and formatted correctly.

### **Comment 19: Verification of Primer Reference Source**

*Line 389 (Reference 21): Please verify this source. Reference [21] is cited for the primers, but it does not appear to be the original source/designer of these sequences. It is better to cite the original methodology papers.*

**Response:** We thank the reviewer for their diligence in ensuring correct attribution. Following your recommendation, we carefully re-verified Reference [21] (*Han et al., 2024*).

Our audit confirmed that these specific primer sequences were originally designed, optimized, and published firsthand by Han et al. within that study; they did not cite any previous literature or external methodology papers for these sequences. Therefore, Reference [21] is the primary, original source for these primers, and retaining this citation is necessary to accurately credit the original designers.
