# Supplementary material for: Molecular diversity and structural profiling of the EmrD efflux pump in multidrug-resistant uropathogenic Escherichia coli
Source: PLoS One. 2026 Nov 11;21(11):e0362259. doi: 10.1371/journal.pone.0362259 (PMC13754136; doi:10.1371/journal.pone.0362259)
Supplement: Supplementary file 4 [file pone.0362259.s006.docx]

### **PART 1: COVER LETTER TO THE ACADEMIC EDITOR**

**Dr. Daniel F. M. Monte, Ph.D.**

**Academic Editor, *PLOS ONE***

**Date:** **September 9, 2026**

**RE: Resubmission of Revised Manuscript (PONE-D-26-16505R1)**

**Title:** Molecular diversity and structural profiling of the EmrD efflux pump in multidrug-resistant uropathogenic *Escherichia coli*

**Manuscript ID:** PONE-D-26-16505R1

Dear Dr. Monte,

On behalf of all co-authors, I am pleased to resubmit our revised manuscript entitled **"Molecular diversity and structural profiling of the EmrD efflux pump in multidrug-resistant uropathogenic *Escherichia coli*"** (Manuscript ID: **PONE-D-26-16505R1**) for consideration for publication as an Original Research Article in *PLOS ONE*.

We express our sincere gratitude to you and the reviewers for the constructive evaluation and thoughtful guidance provided during this second round of review. We are particularly encouraged by Reviewer #2’s complete approval ("All comments have been addressed") and Reviewer #1’s recognition of the substantial improvements made to our interpretive tone, structural clarity, and transparent study limitations.

In response to the remaining minor points raised by Reviewer #1, we have performed a comprehensive revision to strengthen the evolutionary framework of our work without requiring new laboratory experimentation. Specifically:

1. **Expanded Evolutionary Comparator Panel:** We retrieved and incorporated full-length EmrD amino acid sequences from two widely recognized, prototypical UPEC reference genomes—*E. coli* CFT073 (Accession: AAN83032.1) and 536 (Accession: WP_001335494.1)—into our multiple sequence alignment alongside *E. coli* K-12 MG1655. This 13-sequence dataset allowed us to methodologically differentiate baseline UPEC phylogroup traits (e.g., V170A and V304I) from novel, isolate-specific mutations (A173T in isolate DUH 02).
2. **Methodological Reporting & Software Citations:** We explicitly detailed PCR quality controls (routine inclusion of negative non-template controls), refined our operational definition of UPEC (UTI-associated clinical isolates), clarified sequence trimming boundaries (residues 32–345) and added formal literature citations for the primary sequence alignment software utilized (Clustal Omega v1.2.4; Madeira et al., 2024).
3. **Conceptual Framing & Data Alignment:** We updated the subsection title to *"Co-occurrence of emrD variants and 𝛃-lactamase genes"* to remove unsubstantiated biofilm claims, and explicitly uncoupled the plasmid-borne ESBL profile of isolate DUH 11 from its chromosomal *emrD* sequence divergence.

Per *PLOS ONE* submission requirements, the following files have been uploaded separately to the Editorial Manager system:

- **'Response to Reviewers'**: A point-by-point document detailing every response and corresponding manuscript change.
- **'Revised Manuscript with Track Changes'**: A marked-up manuscript file showing all edits highlighted/tracked.
- **'Manuscript'**: A clean, unmarked version of the revised paper.

We confirm that our reference list has been verified for accuracy and completeness, with no retracted papers cited. All authors have reviewed and approved this resubmission, and there are no changes to our financial disclosures or author ORCID details.

Thank you for your time, consideration, and continued editorial stewardship.

Sincerely,

**Dr. Iman H. F. Alnaqshabandy**

Head of the Scientific Research Center

University of Duhok, Duhok, Kurdistan Region, Iraq

Email: iman.fadhiladeen@uod.ac

### **PART 2: RESPONSE TO REVIEWERS**

### **MANUSCRIPT DETAILS**

### **Journal:** *PLOS ONE*

### **Manuscript ID:** PONE-D-26-16505R1

### **Title:** Molecular diversity and structural profiling of the EmrD efflux pump in multidrug-resistant uropathogenic *Escherichia coli*

### **Corresponding Author:** Dr. Iman H. F. Alnaqshabandy

### Dear Academic Editor (Dr. Daniel F. M. Monte) and Reviewers,

### We express our sincere gratitude for the thoughtful, constructive evaluation of our revised manuscript (**PONE-D-26-16505R1**). We are pleased that Reviewer #2 found all previous comments fully addressed, and we deeply appreciate Reviewer #1’s positive recognition of our improved interpretive tone, structural clarity, and transparent study limitations.

### In this second round of revision, we have addressed every remaining methodological and conceptual point raised by Reviewer #1 to further strengthen the evolutionary framework and clarity of our manuscript.

### **Summary of Key Revisions Made in This Version:**

### 1. **Expanded Reference Alignment Dataset (13 Sequences):** We incorporated full-length *emrD* sequences from two prototypical UPEC reference strains—**CFT073** (NCBI Accession: AAN83032.1) and **536** (NCBI Accession: WP_001335494.1)—alongside *E. coli* K-12 MG1655 across our 10 clinical isolates. This dual-comparator approach allowed us to methodologically distinguish baseline, phylogroup-conserved UPEC traits (e.g., V170A and V304I) from novel, isolate-specific mutations (A173T in isolate DUH 02).

### 2. **Methodological Reporting Transparency:** We explicitly detailed PCR quality controls (routine inclusion of negative non-template controls), clarified our operational definition of UPEC (clinical UTI-associated *E. coli*), updated sequence trimming boundaries (analyzed residues 32–345) directly in the Limitations section, and incorporated formal literature citations for the primary alignment software (Clustal Omega v1.2.4).

### 3. **Conceptual Refinement & Data Alignment:** We updated the subsection title to *"Co-occurrence of emrD variants and 𝛃-lactamase genes"* to remove unsubstantiated biofilm claims, and explicitly uncoupled the plasmid-borne ESBL profile of isolate DUH 11 from its chromosomal *emrD* sequence divergence.

### Below is our detailed, point-by-point response to all comments. All corresponding modifications in the manuscript file have been highlighted in the *'Revised Manuscript with Track Changes'* file.

### **I. Responses to Reviewer #1**

**Comment 1: Choice of Comparator Sequence (K-12 vs. Prototypical UPEC References)**

*The most significant concern relates to the reference sequence used for variant calling. Escherichia coli K-12 MG1655 is a well-established comparator in UPEC genomics... but the present analysis is considerably narrower in scope, calling single-nucleotide and amino-acid substitutions within one core gene using K-12 as the sole point of comparison. Because K-12 belongs to phylogroup A while UPEC isolates typically belong to phylogroups B2 or D, some or all of the substitutions identified may reflect general clonal divergence between phylogroups rather than a variant that emerged locally within these isolates... Including the emrD sequence of at least one published, prototypical UPEC genome, such as CFT073 or 536, alongside K-12 in the comparative alignment would allow the authors to distinguish substitutions shared with a UPEC reference... from substitutions unique to individual isolates... This addition requires no new laboratory work and would meaningfully strengthen the evolutionary argument...*

**Response:** We sincerely thank the reviewer for this exceptionally insightful and constructive critique. We completely agree that using *E. coli* K-12 MG1655 (Phylogroup A) as the sole reference sequence introduces potential bias, as lineage-specific baseline variations between phylogroups (e.g., Phylogroup A vs. UPEC-associated Phylogroups B2 or D) could be misidentified as local or novel mutations.

To address this concern directly and resolve this ambiguity methodologically, we expanded our comparative alignment without requiring any new wet-lab experiments. Specifically, we retrieved and incorporated the full-length *emrD* sequences from two widely recognized, prototypical UPEC reference strains—**CFT073** (NCBI Accession: AAN83032.1) and **536** (NCBI Accession: WP_001335494.1)—into our multiple sequence alignment alongside K-12 MG1655.

This dual-comparator approach allowed us to rigorously categorize all identified substitutions into two distinct classes:

1. **Phylogroup-conserved lineages:** Variations shared between our clinical isolates and the prototypical UPEC strains (CFT073/536), which represent broader UPEC backbone traits rather than novel local mutations.
2. **Local lineage variations:** Substitutions unique to individual clinical isolates (absent from both K-12 and the UPEC reference strains), which more plausibly reflect local genetic drift or localized evolutionary pressures.

We have updated our **Materials and methods**, **Results**, **Discussion, Conclusion and Limitation** sections, as well as the alignment figures, to incorporate these references and reflect this strengthened evolutionary classification.

**Location of Changes in the Revised Manuscript:**

- **Materials and methods (Sequence processing and alignment, Lines 151–152):**

“The ten generated consensus sequences along with the reference emrD sequence retrieved from GenBank using E .coli strain k-12 substrain MG1655 and two prototypical uropathogenic E. coli (UPEC) reference strains—CFT073 (Accession: AAN83032.1) and 536 (Accession: WP_001335494.1) were then subjected to multiple sequence alignment (MSA) using Clustal Omega v1.2.4 [23]”

- **Results (Amino acid substitutions and structural localization in EmrD, Lines 259–260 and 263 - 264):**

### “Strain DUH 10 displayed a V170A substitution (Valine to Alanine) in TMH6, which comparative alignment revealed is a baseline polymorphism shared with UPEC reference strain CFT073 (Accession: AAN83032.1). Notably, strain DUH 02 exhibited an A173T substitution (Alanine to Threonine), representing significant physicochemical shift from a non-polar to a polar residue and standing as a novel variant absent in commensal K-12, UPEC reference strains, and wider BLAST datasets”

### “Cross-strain alignment demonstrated that Isoleucine at position 304 (V304I), observed in DUH 04 and DUH 10, is conserved across UPEC reference lineages, including strains CFT073 and 536. While position 304 resides within a conserved region of the consensus, the conservative aliphatic nature of V304I reflects neutral evolutionary divergence characteristic of UPEC isolates rather than a novel destabilizing mutation”

- **Tables (Table 2: Comparative amino acid substitution matrix, Revised Table):**

"Table 2 has been updated to include comparative columns for CFT073 and 536 alongside K-12 MG1655, explicitly flagging whether each detected mutation represents a shared UPEC backbone polymorphism or an isolate-specific substitution."

- **Discussion (Phylogenetic diversity and evolutionary dynamics, Lines 337–341):**

“Importantly, comparative sequence alignment revealed that the V304I substitution is conserved in prototypical UPEC reference strains CFT073 and 536, while V170A matches strain CFT073. Without dN/dS analysis or population-scale genomic data, these substitutions reflect neutral lineage-specific polymorphisms within UPEC rather than adaptive evolution under novel selection pressures.”

### **Discussion (Structural implications of the *emrD* mutations, Lines 345 - 348, 361 - 364)**

### “A pivotal finding of this study is the identification of the V170A mutation in Helix 6 (TM6) and the V304I mutation in Helix 12 (TM12) (Fig. 3). Crucially, cross-strain comparisons demonstrate that V304I represents a baseline UPEC lineage feature shared with both reference strains CFT073 and 536, while V170A is shared specifically with strain CFT073. In contrast, the A173T substitution in strain DUH 02 represents a novel, unique clinical variant absent from all reference strains.”

### “While baseline UPEC lineage polymorphisms (V170A and V304I) were present in 4% and 8% of our initial screening BLAST dataset, respectively, the novel A173T variant remains unobserved across global reference datasets, highlighting its unique distribution in clinical isolate DUH 02.”

### **Limitations, Lines 385 - 386**

### “Second, while we identified non-synonymous amino acid substitutions (the novel A173T variant alongside UPEC lineage features V170A and V304I),”

### **Conclusion, Lines 397 - 398**

### “The identification of a novel structural mutation in TMH6 (A173T in strain DUH 02), alongside baseline UPEC lineage polymorphisms (V170A and V304I), supports a model of "induced fit" evolution in MFS transporters, wherein localized structural variations optimize transport dynamics without compromising overall scaffold stability.”

### **Comment 2: Reference Comparators (K-12 vs. UPEC References) and Diversity of the 25 BLAST Sequences**

### *The most significant concern relates to the reference sequence used for variant calling... Including the emrD sequence of at least one published, prototypical UPEC genome, such as CFT073 or 536, alongside K-12 in the comparative alignment would allow the authors to distinguish substitutions shared with a UPEC reference but absent from K-12... A related concern applies to the set of 25 reference sequences used to calculate how rare or common each substitution is... Since the claims of rarity for the A173T and V304I substitutions rest directly on this reference set, a more phylogenetically stratified sampling approach could yield meaningfully different frequencies, and this should either be justified explicitly or repeated with a more diverse comparator set.*

### **Response:**

### We thank the reviewer for this crucial insight. We fully agree that relying solely on *E. coli* K-12 MG1655 introduces ambiguity when attempting to differentiate baseline UPEC phylogroup divergence from novel, isolate-specific variants.

### To address this, **we updated our multiple sequence alignment (MSA) in the primary sequence processing workflow to directly include two prototypical, published UPEC reference genomes—*E. coli* CFT073 (Accession: AAN83032.1) and 536 (Accession: WP_001335494.1)—alongside K-12 MG1655 across our 10 clinical isolates.**

### This addition cleanly resolves the analytical ambiguity:

### 1. **Differentiating UPEC Lineage Traits:** Aligning against CFT073 and 536 demonstrated that substitutions such as **V304I** (and **V170A** in CFT073) are conserved in prototypical UPEC reference genomes, establishing them as neutral, baseline UPEC lineage background polymorphisms rather than local, isolate-specific mutations.

### 2. **Confirming Isolate-Specific Variants:** Conversely, the **A173T** substitution in isolate DUH 02 remained absent in K-12 MG1655, CFT073, and 536, confirming it as a unique, isolate-specific variant within our clinical cohort.

### Regarding the **25 GenBank reference sequences**, we appreciate the opportunity to clarify that their role is distinct from our primary alignment controls (CFT073 and 536). In our revised ***In Silico* conservation and frequency analysis** section, we explicitly describe these 25 sequences as a curated, non-redundant global panel retrieved via BLASTp (≥98\% query coverage, ≥95\% identity) across diverse pathotypes and geographic origins. This global panel was utilized specifically to calculate substitution frequencies (e.g., assessing the rarity of A173T and V304I) and evaluate domain conservation without cluttering or overcrowding the phylogenetic alignment with hundreds of nearly identical sequences.

### We have updated both corresponding subsections in the **Materials and methods** as well as the **Discussion** to reflect these additions cleanly without duplication.

### **Location of Changes in the Revised Manuscript:**

### **Materials and methods (*In Silico conservation and frequency analysis, Lines 173 - 179*):** "To evaluate the broader evolutionary conservation of the identified EmrD variants, a protein BLAST (BLASTp) search was conducted against the NCBI non-redundant (nr) protein database using the *E. coli* K-12 EmrD sequence as a query. **A curated panel of 25 representative *E. coli* reference sequences displaying high query coverage (**≥ **98%) and sequence identity (**≥ **95%) across diverse pathotypes and geographic origins was retrieved. This non-redundant global dataset was used to calculate the frequency of specific amino acid substitutions (e.g., A173T and V304I) and to determine whether observed mutations occurred within conserved transmembrane helices or variable loop regions.**"

### **Comment 3: Methodological Reporting Gaps (PCR Controls, UPEC Definition, MLST, and Sequence Coverage)**

### *Several smaller methodological gaps compound these concerns. No positive or negative PCR controls are reported for any of the assays, which is particularly relevant given that the beta-lactamase genes were identified by PCR alone and never sequenced. The manuscript speculates that the divergent isolate DUH 11 may belong to a distinct E. coli phylogroup, but no phylotyping or MLST was performed to test this directly, leaving the claim untestable with the data presented. Similarly, all isolates are described as UPEC throughout the manuscript based solely on their clinical source, without confirmation by any UPEC-associated virulence markers, which technically makes them E. coli isolates associated with urinary tract infection rather than confirmed UPEC in the pathotype sense. Finally, the S2 figure legend notes that roughly the first 31 amino acids of each clinical isolate were not sequenced, meaning that variation in this region could not have been detected in any isolate; this gap is directly relevant to the study's central claim of having characterized emrD diversity and should be carried into the Limitations section rather than left in a figure legend alone.*

### **Response:**

### We thank the reviewer for highlighting these key methodological and reporting details. We fully agree that providing explicit information regarding PCR controls, isolate classification criteria, typing scope, and sequence coverage boundaries is essential for complete scientific rigor and transparency. We have addressed all four points through focused, minimal revisions in both the Materials and methods and Discussion sections:

### 1. **PCR Quality Controls and Assay Validation:** We clarified that negative non-template controls (NTC; nuclease-free water) were routinely included in every PCR batch to confirm reagent purity and rule out false-positive contamination. Additionally, species identity for all isolates had been previously confirmed at the molecular level (housekeeping gene PCR) and biochemically (BD Phoenix automated system) during prior thesis work.

### 2. **UPEC Terminology and Definition:** We clarified that isolates were operationally defined based on clinical diagnostic criteria ( 10^5^ CFU/mL pure culture from symptomatic UTI patients) rather than molecular pathotyping. To maintain strict scientific accuracy, we updated the text to refer to them as "clinical UTI-associated *E. coli* (operationally categorized as UPEC)" and explicitly acknowledged the bounded scope in the Limitations section.

### 3. **Phylotyping and MLST Framing:** We refined the discussion of isolate DUH 11 to avoid speculative claims about specific sequence types or phylogroups without MLST data, framing its divergence strictly as potential chromosomal heterogeneity or silent background variation relative to reference strains.

### 4. **Sequence Coverage Boundaries:** We updated the sequence processing description and the Study Limitations section to reflect the exact analyzed range (residues 32–345), explicitly acknowledging that terminal primer quality trimming left 31 N-terminal and 50 C-terminal amino acids uncharacterized across all isolates.

### **Location of Changes in the Revised Manuscript:**

### **Materials and methods (*Bacterial strains and DNA samples*, Lines 93–96):** "Clinical *Escherichia coli* isolates were recovered during routine diagnostic procedures from patients diagnosed with urinary tract infections (UTIs) at Azadi Teaching Hospital, Duhok, Kurdistan Region, Iraq. In this study, isolates are operationally defined as uropathogenic *E. coli* (UPEC) based on clinical source criteria (recovery as significant monocultures 10^5^ CFU/mL from symptomatic UTI patients). Species identity was previously confirmed at the molecular level via housekeeping gene PCR and biochemically using the BD Phoenix automated microbiology system during a prior master’s research project [20]. The isolates were selected for the current molecular characterization based on their extensive multidrug-resistant (MDR) profiles."

### **Materials and methods (*Molecular detection of β-Lactamase and the emrD gene*, Lines 126–128):** "Amplification of *emrD* and 𝛃-lactamase genes (*bla_NDM_*, *bla_SHV_*, *bla_TEM_*, *bla_CTX-M-1_* and *bla_CTX-M-9_*) was performed using specific primers... To monitor for reagent contamination and non-specific amplification, a negative non-template control (NTC; replacing DNA template with nuclease-free water) was routinely included in every PCR batch, consistently demonstrating no non-specific amplification."

### **Materials and methods (*Sequence processing and alignment*, Lines 145–146):** "Sequence chromatograms were visually inspected and quality-trimmed to remove low-quality bases at the 5' and 3' ends using the Benchling webserver. End-trimming of primer-binding regions trimmed ~31 N-terminal and ~50 C-terminal amino acids, leaving residues 32–345 for analysis across all isolates. Sequences were retained only if Phred quality scores exceeded 30 (99.9% accuracy)."

### **Discussion (*Phylogenetic diversity and evolutionary dynamics*, Lines 330–334):** "In contrast, the extensive branch length observed for strain DUH 11 highlights a divergent evolutionary trajectory. This outlier status likely reflects the accumulation of silent mutations or a distinct phylogenetic background (e.g., a different *E. coli* phylogroup), similar to the chromosomal heterogeneity recently described in UPEC isolates from Uganda by Maghembe et al. (2024) [19]."

### **Limitations, Lines 382–388):** "Third, the scope was bounded by clinical operational categorization of UPEC rather than molecular virulence or MLST/phylogroup profiling, and 𝛃-lactamase genes were characterized by PCR screen alone. Finally, primer end-trimming restricted analyzed sequence coverage to residues 32–345, leaving potential variation in the first 31 N-terminal and last 50 C-terminal amino acids unevaluated."

**Comment 4:**

*"...the subsection addressing co-resistance and biofilm potential does not present any biofilm data, and the paragraph closes by citing general literature on the association between efflux regulation and biofilm formation without connecting it back to the study's own findings; this reads as an unfinished thought... Elsewhere, the manuscript places DUH 11's blaSHV-positive status next to its description as the most phylogenetically divergent isolate without establishing any mechanistic or statistical link..."*

**Response:**

We thank the reviewer for these perceptive observations regarding section titles and data framing.

We have addressed both points in the revised manuscript as follows:

1. **Subsection Retitling:** To ensure the heading accurately reflects the content and does not imply empirical biofilm testing, **we updated the subsection title to "Co-occurrence of *emrD* variants and 𝛃-lactamase genes."** We retain the brief literature citation [24] strictly as general background context regarding efflux pump regulatory networks rather than as a claim of phenotypic biofilm characterization in our isolates.
2. **Uncoupling DUH 11 ESBL Profile, Lines 334 - 336:** We have added in the Discussion section “**Phylogenetic diversity and evolutionary dynamics**” that **“We emphasize that the *bla_SHV_*-positive status of DUH 11 (along with *bla_CTX-M-1_* and *bla_TEM_*) is plasmid-borne and uncoupled from its chromosomal sequence divergence.”** This restructuring removes any implied causal link between its mobile resistance genotype and its phylogenetic position.
