# Supplementary material for: Molecular diversity and structural profiling of the EmrD efflux pump in multidrug-resistant uropathogenic Escherichia coli
Source: PLoS One. 2026 Nov 11;21(11):e0362259. doi: 10.1371/journal.pone.0362259 (PMC13754136; doi:10.1371/journal.pone.0362259)
Supplement: Peer review history

# Peer review: Decision Letter 0

**DOI:** 10.1371/journal.pone.0362259.r001

**Authors:** Daniel F. M. Monte

2 Jun 2026

Dear Dr. Alnaqshabandy,

Thank you for submitting your manuscript to PLOS ONE. After careful consideration, we feel that it has merit but does not fully meet PLOS ONE’s publication criteria as it currently stands. Therefore, we invite you to submit a revised version of the manuscript that addresses the points raised during the review process.

Please submit your revised manuscript by Jul 17 2026 11:59PM. If you will need more time than this to complete your revisions, please reply to this message or contact the journal office at plosone@plos.org. When you're ready to submit your revision, log on to https://www.editorialmanager.com/pone/ and select the 'Submissions Needing Revision' folder to locate your manuscript file.

- A letter that responds to each point raised by the academic editor and reviewer(s). You should upload this letter as a separate file labeled 'Response to Reviewers'.
- A marked-up copy of your manuscript that highlights changes made to the original version. You should upload this as a separate file labeled 'Revised Manuscript with Track Changes'.
- An unmarked version of your revised paper without tracked changes. You should upload this as a separate file labeled 'Manuscript'.

If you would like to make changes to your financial disclosure, please include your updated statement in your cover letter. Guidelines for resubmitting your figure files are available below the reviewer comments at the end of this letter.

If applicable, we recommend that you deposit your laboratory protocols in protocols.io to enhance the reproducibility of your results. Protocols.io assigns your protocol its own identifier (DOI) so that it can be cited independently in the future. For instructions see: https://journals.plos.org/plosone/s/submission-guidelines#loc-laboratory-protocols. Additionally, PLOS ONE offers an option for publishing peer-reviewed Lab Protocol articles, which describe protocols hosted on protocols.io. Read more information on sharing protocols at https://plos.org/protocols?utm_medium=editorial-email&utm_source=authorletters&utm_campaign=protocols.

As the corresponding author, your ORCID iD is verified in the submission system and will appear in the published article. PLOS supports the use of ORCID, and we encourage all coauthors to register for an ORCID iD and use it as well. Please encourage your coauthors to verify their ORCID iD within the submission system before final acceptance, as unverified ORCID iDs will not appear in the published article. *Only*  the individual author can complete the verification step; PLOS staff *cannot*  verify ORCID iDs on behalf of authors.

We look forward to receiving your revised manuscript.

Kind regards,

Daniel F. M. Monte, Ph.D.

Academic Editor

PLOS One

Journal Requirements:

When submitting your revision, we need you to address these additional requirements.

1. Please ensure that your manuscript meets PLOS ONE's style requirements, including those for file naming. The PLOS ONE style templates can be found at

https://journals.plos.org/plosone/s/file?id=wjVg/PLOSOne_formatting_sample_main_body.pdf and

https://journals.plos.org/plosone/s/file?id=ba62/PLOSOne_formatting_sample_title_authors_affiliations.pdf

2. Please note that your Data Availability Statement is currently missing the accession number of each dataset can’t access. If your manuscript is accepted for publication, you will be asked to provide these details on a very short timeline. We therefore suggest that you provide this information now, though we will not hold up the peer review process if you are unable.

3. PLOS ONE now requires that authors provide the original uncropped and unadjusted images underlying all blot or gel results reported in a submission’s figures or Supporting Information files. This policy and the journal’s other requirements for blot/gel reporting and figure preparation are described in detail at https://journals.plos.org/plosone/s/figures#loc-blot-and-gel-reporting-requirements and https://journals.plos.org/plosone/s/figures#loc-preparing-figures-from-image-files. When you submit your revised manuscript, please ensure that your figures adhere fully to these guidelines and provide the original underlying images for all blot or gel data reported in your submission. See the following link for instructions on providing the original image data: https://journals.plos.org/plosone/s/figures#loc-original-images-for-blots-and-gels.

In your cover letter, please note whether your blot/gel image data are in Supporting Information or posted at a public data repository, provide the repository URL if relevant, and provide specific details as to which raw blot/gel images, if any, are not available. Email us at plosone@plos.org if you have any questions.

4. If the reviewer comments include a recommendation to cite specific previously published works, please review and evaluate these publications to determine whether they are relevant and should be cited. There is no requirement to cite these works unless the editor has indicated otherwise.

Additional Editor Comments:

Thank you for submitting your manuscript. The reviewer acknowledges the relevance of the topic and the value of investigating the molecular diversity of the emrD efflux transporter in MDR UPEC isolates. The integration of sequence analysis, phylogenetic assessment, and structural mapping is a strength of the study, and the data provide useful descriptive information from an underrepresented geographic region.

However, substantial revisions are required. The main concern is that several conclusions extend beyond the evidence presented. While the study identifies emrD variants and maps them to structurally relevant regions, no functional experiments were performed to demonstrate effects on efflux activity, antimicrobial resistance, substrate specificity, virulence, or adaptation. Therefore, interpretations suggesting adaptive evolution, altered transporter function, or direct contributions to multidrug resistance should be significantly moderated and presented as hypotheses rather than conclusions.

The discussion should more clearly distinguish between observations supported by the data and speculative interpretations. In addition, the limitations of the study—including the small sample size, lack of susceptible comparator strains, limited phylogenetic context, and absence of functional validation—should be more explicitly acknowledged. The phylogenetic and evolutionary interpretations should also be revised to avoid unsupported inferences regarding selection, adaptation, or dissemination.

Overall, the manuscript has merit as an exploratory and hypothesis-generating study, but its conclusions require substantial revision to align with the data presented. The authors should carefully address all reviewer comments and provide a detailed point-by-point response in the revised submission.

[Note: HTML markup is below. Please do not edit.]

Reviewers' comments:

Reviewer's Responses to Questions

**Comments to the Author**

1. Is the manuscript technically sound, and do the data support the conclusions?

Reviewer #1: Partly

Reviewer #2: Yes

**********

2. Has the statistical analysis been performed appropriately and rigorously?

Reviewer #1: N/A

Reviewer #2: Yes

**********

3. Have the authors made all data underlying the findings in their manuscript fully available?

The PLOS Data policy

Reviewer #1: Yes

Reviewer #2: No

**********

4. Is the manuscript presented in an intelligible fashion and written in standard English?

Reviewer #1: Yes

Reviewer #2: Yes

**********

Reviewer #1: The manuscript presents a relevant and potentially interesting proposal by investigating the molecular diversity of the EmrD efflux transporter in clinical MDR UPEC isolates, particularly through the integration of phylogenetic analysis, mutation identification, and structural contextualisation. The overall rationale is coherent and well aligned with the current antimicrobial resistance landscape in uropathogenic Escherichia coli. The Introduction adequately contextualises efflux systems within the MFS superfamily and appropriately positions EmrD as a comparatively underexplored component relative to more extensively studied systems such as AcrAB-TolC. Moreover, the study is logically organised, and the integration of molecular and structural analyses constitutes the strongest aspect of the work.

Despite these strengths, the manuscript suffers from a recurring and substantial issue: it consistently extrapolates beyond what the data actually demonstrate. In several instances, the text transforms plausible hypotheses into near-assertive interpretations, particularly when suggesting that mutations within TMH6 and TMH12 may alter transport efficiency, conformational plasticity, adaptation to clinical environments, or even contribute directly to multidrug resistance. The study demonstrates only the universal presence of emrD, the existence of sequence variation, and the structural localisation of these substitutions within relevant transmembrane regions. No functional experiments were conducted to demonstrate altered efflux activity, changes in substrate specificity, differences in MIC values, altered gene expression, or any phenotypic consequence associated with the identified variants.

This limitation is particularly important because the manuscript implicitly constructs a mechanistic chain in which structural mutations would lead to conformational changes, subsequently modifying substrate export efficiency and ultimately contributing to adaptation under antibiotic pressure. None of these intermediate steps are experimentally validated. As a result, the text frequently implies causality where only observational correlation exists. This concern is amplified by the fact that emrD is a chromosomal core gene broadly distributed across E. coli, including fully susceptible strains. Consequently, the mere presence of emrD in MDR isolates does not support conclusions regarding its functional contribution to the resistance phenotype.

Another major concern relates to the evolutionary interpretation of the identified mutations. The manuscript repeatedly describes rare variants as “specialised adaptations” or evidence of “structural fine-tuning under antibiotic pressure”, yet no robust evolutionary analyses were performed to support such claims. There is no assessment of positive selection, dN/dS ratios, codon-level evolutionary pressure, population structure, or broader phylogenetic context using large-scale genomic datasets. The rarity of a mutation does not, in itself, imply adaptive significance. The observed substitutions may simply reflect neutral drift, lineage-specific polymorphism, chromosomal background variation, or stochastic diversification within local UPEC populations.

The structural discussion is conceptually interesting, particularly in linking TMH6 and TMH12 to the “rocker-switch” mechanism characteristic of MFS transporters. Nevertheless, the manuscript repeatedly exceeds the interpretative limits permitted by positional structural mapping alone. For example, the V304I substitution is biochemically highly conservative, involving hydrophobic branched-chain residues with similar physicochemical properties. Even the A173T mutation, although potentially more relevant due to its polar shift, remains entirely hypothetical in functional terms. The manuscript repeatedly infers altered conformational flexibility, modified cavity architecture, or altered substrate recognition despite the absence of molecular dynamics simulations, docking analyses, stability calculations, or any direct structural modelling beyond residue localisation.

The phylogenetic analysis also requires more cautious interpretation. The study includes only ten clinical isolates and a highly limited comparative dataset. The identified clustering patterns are likely to reflect broader clonal relationships among bacterial lineages rather than adaptive selection acting specifically upon emrD. Without genome-wide phylogenetic context, it is impossible to distinguish gene-specific adaptation from simple chromosomal inheritance. Furthermore, the extremely small sample size severely limits any meaningful inference regarding mutation frequency, evolutionary dynamics, or regional dissemination patterns.

From a methodological standpoint, the work is acceptable as an exploratory pilot study but falls considerably short of the current state-of-the-art required to support mechanistic conclusions. The absence of susceptible control strains is a major limitation, as it prevents any assessment of whether the identified variants are enriched among MDR isolates. Similarly, no expression analyses were performed, such as RT-qPCR, which could at least have addressed whether regulatory differences accompany the coding sequence variation. Functional assays such as ethidium bromide accumulation, efflux inhibition assays, MIC comparisons, site-directed mutagenesis, or complementation experiments would be essential to validate the proposed functional implications of the mutations identified.

The Discussion section is arguably the most problematic component of the manuscript. Although generally well written and supported by relevant literature on MFS transporters, it repeatedly inflates the biological implications of the findings. The text attributes potential roles to EmrD in virulence, intracellular survival, host adaptation, and biofilm formation, despite none of these features having been experimentally investigated in this study. Moreover, the cited literature often concerns MFS efflux systems broadly rather than EmrD specifically in the analysed strains. The manuscript therefore transfers general properties of the superfamily directly onto the specific variants identified here, which is not sufficiently justified.

In terms of novelty, the study possesses moderate merit as a regional exploratory survey. The identification and structural contextualisation of emrD variants in Iraqi MDR UPEC isolates may hold descriptive and epidemiological value, particularly given the relative underrepresentation of such populations in the literature. However, the majority of the work remains fundamentally descriptive. The principal contribution lies in variant detection and structural localisation rather than the demonstration of novel resistance mechanisms. Consequently, the manuscript is more appropriately framed as a preliminary molecular diversity study than as evidence for adaptive structural evolution of EmrD associated with multidrug resistance.

The figures are broadly coherent with the manuscript text but present several limitations. Figure 1 suffers from poor visual quality and insufficient contrast. Figure 2 has limited resolution and poorly interpretable bootstrap support, whilst Figure 3, although conceptually the strongest, represents only positional mapping rather than rigorous structural analysis. The figure legends generally correspond well to the manuscript; however, in some cases they reinforce mechanistic interpretations that are not adequately supported by the data.

Overall, the manuscript contains potentially useful observations and an interesting attempt to integrate genetic diversity with structural protein analysis. Nevertheless, the conclusions require substantial moderation. The study should be reframed as an exploratory and hypothesis-generating analysis rather than as evidence of functional adaptive evolution linked to multidrug resistance. The primary revision required is a clearer distinction between what has actually been demonstrated and what remains speculative. Without additional functional validation, statements relating to altered efflux efficiency, adaptation under antibiotic pressure, virulence contribution, or structural specialisation should be reformulated using considerably more cautious and hypothetical language.

Reviewer #2: General Comments and Analysis: This manuscript provides an interesting genomic and structural characterization of the emrD efflux pump in multidrug-resistant (MDR) Uropathogenic Escherichia coli (UPEC) isolates from the Kurdistan region. The study is highly relevant as it bridges the gap between traditional molecular epidemiology (detection of bla genes) and structural bioinformatics. By identifying specific mutations in the transmembrane helices (TMH6 and TMH12) and correlating them with phylogenetic lineages, the authors offer a sophisticated view of bacterial adaptation. The use of protein modeling (PDB: 2GFP) to hypothesize on the "induced fit" mechanism adds significant value. However, several technical inconsistencies, nomenclature issues, and formatting errors must be addressed to meet the standard for international publication.

Specific Suggestions and Comments

Title and Affiliations

Affiliation Mapping: Please clarify why the affiliations are indicated with two different numbers if they refer to the same institution. This should be consolidated to avoid confusion.

Introduction and Background

Line 68: “membrane [15,16]”. Ensure there is a space before the brackets, if required by the journal style.

Line 73: Extra letters “Uropathogenic”.

Line 74: Grammar correction: Change to "...responsible for 75% of uncomplicated..." to ensure clarity.

Line 103: Regarding the mention of cloxacillin: It is important to note that E. coli is inherently resistant to cloxacillin. Reporting this as an "acquired resistance" or a significant finding might be seen as a lack of microbiological context.

Materials and Methods

Line 114 (Primer Concentration): There is a potential discrepancy in the reaction volume. You mention using 2 µL of a 5 µM stock, which results in 10 pmol per reaction. Please verify if the intended amount was 5 pmol (absolute amount) or if the stock concentration was different.

Line 119 (Table 1 & References): * It is highly recommended to merge the primer data into a single, cohesive table. Currently, blaNDM appears twice (positions 1 and 6).

Reference [21] must be cited in the main text. Example: "The primers used for β-lactamase gene detection were obtained from previous studies [21] and are summarized in Table 1."

Line 135: Specify the exact strain of E. coli K-12 used (e.g., MG1655, W3110).

Line 136: Define the acronym upon first use: MAFFT (Multiple Alignment using Fast Fourier Transform).

Line 139: Accession Numbers: I was unable to locate the mentioned accession numbers in the NCBI database. These must be public and searchable before publication.

Line 151 (Reference Sequences): Please provide a rationale for selecting exactly 25 reference sequences. Were these selected to represent different phylogroups, or were they simply the top 25 hits from a BLAST search?

Line 152 (Table Numbering): Table 3 is cited before Table 2. Tables must be numbered sequentially according to their appearance in the text.

Results

Line 156: Suggested phrasing: "...exhibited a uniform multidrug-resistant (MDR) …"

Line 157: Mentioning "100% resistance" to cloxacillin is technically accurate but clinically irrelevant for E. coli. I suggest removing or de-emphasizing this.

Line 165 (PCR Product Size): In Table 1, the expected size is 1,076 bp. However, the text mentions approximately 1,200 bp. This discrepancy (over 100 bp) needs to be explained or corrected to ensure the integrity of the PCR results.

Line 200 (Character Rendering): The symbol “ ” (Beta) appears as a “Ghost Character” in my PDF reader. Please check if this is a rendering error or if there is actually a problem.

Line 204 (Strain Nomenclature): Standardize the strain IDs throughout the manuscript. The text alternates between space and hyphen (e.g., "DUH 04" vs. "DUH-04").

Line 205 (Phylogeny): There is a contradiction regarding the "basal" strain. Earlier, DUH 11 was identified as the divergent/basal outlier, but here DUH 01 is mentioned.

References

Line 341 (Zotero/Citations): Before submission, ensure you "Unlink Citations" if using Zotero. Also, standardize journal names: either use full names or standard ISO abbreviations consistently for all entries.

Line 354: There is a repeated title in section 7. Please remove the duplication.

Line 389 (Reference 21): Please verify this source. Reference [21] is cited for the primers, but it does not appear to be the original source/designer of these sequences. It is better to cite the original methodology papers.

**********

what does this mean?). If published, this will include your full peer review and any attached files.

If you choose “no”, your identity will remain anonymous but your review may still be made public.

**Do you want your identity to be public for this peer review?**  For information about this choice, including consent withdrawal, please see our Privacy Policy

Reviewer #1: No

Reviewer #2: **Yes:**  Alan Douglas de Lima Rocha

**********

[NOTE: If reviewer comments were submitted as an attachment file, they will be attached to this email and accessible via the submission site. Please log into your account, locate the manuscript record, and check for the action link "View Attachments". If this link does not appear, there are no attachment files.]

To ensure your figures meet our technical requirements, please review our figure guidelines: https://journals.plos.org/plosone/s/figures

You may also use PLOS’s free figure tool, NAAS, to help you prepare publication quality figures: https://journals.plos.org/plosone/s/figures#loc-tools-for-figure-preparation.

NAAS will assess whether your figures meet our technical requirements by comparing each figure against our figure specifications.

# Author comment: Author response to Decision Letter 1

**DOI:** 10.1371/journal.pone.0362259.r002

29 Jul 2026

Dear Academic Editor Dr. Daniel F. M. Monte and Reviewers,

Thank you very much for your highly constructive and encouraging feedback on our manuscript (PONE-D-26-16505). We have taken all comments into serious consideration and have thoroughly revised our manuscript to address every point raised.

Due to the length and detailed formatting of our point-by-point responses (including line-number references and blockquotes), we have uploaded our complete, detailed "Response to Reviewers" as a separate document.

Below is a brief summary of the key revisions we have implemented:

Moderated Interpretative Claims (Editor & Reviewer 1): We have significantly moderated our evolutionary and structural conclusions—shifting them from definitive claims to exploratory hypotheses—to accurately reflect the pilot nature of this survey.

Dedicated Study Limitations (Editor & Reviewer 1): We have added a dedicated "Study Limitations" section explicitly addressing the pilot nature of the study, the small sample size (n=10), and the lack of molecular dynamics simulations, RT-qPCR transcript quantification, or site-directed mutagenesis to kinetically validate the V170A/V304I variants.

Correction of Nomenclature & Technical Formatting (Reviewer 2): We have meticulously addressed all technical points raised by Reviewer 2 across the manuscript's 19 comments. This includes standardizing our strain naming convention (e.g., using "DUH 04" consistently), correcting the phylogenetic branch descriptions (clarifying the basal position of DUH 11), resolving font-rendering issues for the symbol "β", adjusting PCR primer/volume calculations, and unlinking Zotero citation metadata.

Data Availability & Integrity (Editor & Reviewer 2): All 10 generated nucleotide sequences are now fully public and searchable in the NCBI GenBank database (accession numbers PZ196343 through PZ196352). Additionally, we have provided original, uncropped gel images under "S1_Raw_Images".

We hope these extensive revisions and clarifications make our manuscript fully suitable for publication in PLOS ONE.

Please refer to our uploaded "Response to Reviewers" file for the complete, word-for-word responses to every individual comment.

Sincerely,

Dr. Iman H. F. Alnaqshabandy

University of Duhok

Attachment Submitted filename: Response to Reviewers.docx

# Peer review: Decision Letter 1

**DOI:** 10.1371/journal.pone.0362259.r003

**Authors:** Daniel F. M. Monte

19 Aug 2026

Dear Dr. Alnaqshabandy,

Thank you for submitting your manuscript to PLOS One. After careful consideration, we feel that it has merit but does not fully meet PLOS One’s publication criteria as it currently stands. Therefore, we invite you to submit a revised version of the manuscript that addresses the points raised during the review process.

Please submit your revised manuscript by Oct 03 2026 11:59PM. If you will need more time than this to complete your revisions, please reply to this message or contact the journal office at plosone@plos.org. When you're ready to submit your revision, log on to https://www.editorialmanager.com/pone/ and select the 'Submissions Needing Revision' folder to locate your manuscript file.

- A letter that responds to each point raised by the academic editor and reviewer(s). You should upload this letter as a separate file labeled 'Response to Reviewers'.
- A marked-up copy of your manuscript that highlights changes made to the original version. You should upload this as a separate file labeled 'Revised Manuscript with Track Changes'.
- An unmarked version of your revised paper without tracked changes. You should upload this as a separate file labeled 'Manuscript'.

If you would like to make changes to your financial disclosure, please include your updated statement in your cover letter. Guidelines for resubmitting your figure files are available below the reviewer comments at the end of this letter.

If applicable, we recommend that you deposit your laboratory protocols in protocols.io to enhance the reproducibility of your results. Protocols.io assigns your protocol its own identifier (DOI) so that it can be cited independently in the future. For instructions see: https://journals.plos.org/plosone/s/submission-guidelines#loc-laboratory-protocols. Additionally, PLOS One offers an option for publishing peer-reviewed Lab Protocol articles, which describe protocols hosted on protocols.io. Read more information on sharing protocols at https://plos.org/protocols?utm_medium=editorial-email&utm_source=authorletters&utm_campaign=protocols.

As the corresponding author, your ORCID iD is verified in the submission system and will appear in the published article. PLOS supports the use of ORCID, and we encourage all coauthors to register for an ORCID iD and use it as well. Please encourage your coauthors to verify their ORCID iD within the submission system before final acceptance, as unverified ORCID iDs will not appear in the published article. *Only*  the individual author can complete the verification step; PLOS staff *cannot*  verify ORCID iDs on behalf of authors.

We look forward to receiving your revised manuscript.

Kind regards,

Daniel F. M. Monte, Ph.D.

Academic Editor

PLOS One

Journal Requirements:

If the reviewer comments include a recommendation to cite specific previously published works, please review and evaluate these publications to determine whether they are relevant and should be cited. There is no requirement to cite these works unless the editor has indicated otherwise.

Please review your reference list to ensure that it is complete and correct. If you have cited papers that have been retracted, please include the rationale for doing so in the manuscript text, or remove these references and replace them with relevant current references. Any changes to the reference list should be mentioned in the rebuttal letter that accompanies your revised manuscript. If you need to cite a retracted article, indicate the article’s retracted status in the References list and also include a citation and full reference for the retraction notice.

**Additional Editor Comments:**

Please, address the comments point-by-point.

[Note: HTML markup is below. Please do not edit.]

Reviewers' comments:

Reviewer's Responses to Questions

**Comments to the Author**

Reviewer #1: (No Response)

Reviewer #2: All comments have been addressed

**********

2. Is the manuscript technically sound, and do the data support the conclusions?

Reviewer #1: Partly

Reviewer #2: Yes

**********

3. Has the statistical analysis been performed appropriately and rigorously?

Reviewer #1: N/A

Reviewer #2: Yes

**********

4. Have the authors made all data underlying the findings in their manuscript fully available?

The PLOS Data policy

Reviewer #1: Yes

Reviewer #2: Yes

**********

5. Is the manuscript presented in an intelligible fashion and written in standard English?

Reviewer #1: Yes

Reviewer #2: Yes

**********

Reviewer #1: The manuscript presents a coherent and well-organized pilot study integrating molecular detection, sequencing, phylogenetic analysis, and structural mapping to characterize genomic diversity in the emrD efflux pump among ten clinical MDR UPEC isolates. Following the previous round of revision, the interpretive tone has been substantially improved, with speculative and mechanistic language now more consistently distinguished from observation, and the newly added Limitations section appropriately acknowledges the pilot nature of the study and the absence of functional validation. Despite these improvements, several methodological and conceptual issues remain that constrain how confidently the reported findings can be interpreted.

The most significant concern relates to the reference sequence used for variant calling. Escherichia coli K-12 MG1655 is a well-established comparator in UPEC genomics for whole-genome comparisons or virulence-related phenotypic assays, where it serves as the species' neutral genomic backbone, but the present analysis is considerably narrower in scope, calling single-nucleotide and amino-acid substitutions within one core gene using K-12 as the sole point of comparison. Because K-12 belongs to phylogroup A while UPEC isolates typically belong to phylogroups B2 or D, some or all of the substitutions identified may reflect general clonal divergence between phylogroups rather than a variant that emerged locally within these isolates. The authors appear to recognize this ambiguity themselves in the Results, noting that it is unclear whether the observed variation reflects pressures acting specifically on emrD or simply differences in broader clonal background, yet this uncertainty is never resolved methodologically. Including the emrD sequence of at least one published, prototypical UPEC genome, such as CFT073 or 536, alongside K-12 in the comparative alignment would allow the authors to distinguish substitutions shared with a UPEC reference but absent from K-12, which would more plausibly reflect UPEC-associated divergence, from substitutions unique to individual isolates and absent from both references, which would be more consistent with local lineage drift. This addition requires no new laboratory work and would meaningfully strengthen the evolutionary argument the Discussion is attempting to build.

A related concern applies to the set of 25 reference sequences used to calculate how rare or common each substitution is. Because these were selected as the top BLASTp hits by identity and coverage, the resulting set is likely to be highly similar both to the query and to one another, which is not equivalent to a representative sample of global E. coli diversity. Since the claims of rarity for the A173T and V304I substitutions rest directly on this reference set, a more phylogenetically stratified sampling approach could yield meaningfully different frequencies, and this should either be justified explicitly or repeated with a more diverse comparator set.

Several smaller methodological gaps compound these concerns. No positive or negative PCR controls are reported for any of the assays, which is particularly relevant given that the beta-lactamase genes were identified by PCR alone and never sequenced. The manuscript speculates that the divergent isolate DUH 11 may belong to a distinct E. coli phylogroup, but no phylotyping or MLST was performed to test this directly, leaving the claim untestable with the data presented. Similarly, all isolates are described as UPEC throughout the manuscript based solely on their clinical source, without confirmation by any UPEC-associated virulence markers, which technically makes them E. coli isolates associated with urinary tract infection rather than confirmed UPEC in the pathotype sense. Finally, the S2 figure legend notes that roughly the first 31 amino acids of each clinical isolate were not sequenced, meaning that variation in this region could not have been detected in any isolate; this gap is directly relevant to the study's central claim of having characterized emrD diversity and should be carried into the Limitations section rather than left in a figure legend alone.

On the conceptual side, the subsection addressing co-resistance and biofilm potential does not present any biofilm data, and the paragraph closes by citing general literature on the association between efflux regulation and biofilm formation without connecting it back to the study's own findings; this reads as an unfinished thought that likely needs either a clearer framing as background context or removal of the biofilm framing altogether. Elsewhere, the manuscript places DUH 11's blaSHV-positive status next to its description as the most phylogenetically divergent isolate without establishing any mechanistic or statistical link between the two, which risks implying a connection to the reader that the data do not actually support.

Reviewer #2: Thank you for thoroughly addressing all questions and making the necessary corrections to the manuscript. The revisions are clear, and I have no further concerns regarding research or publication ethics.

**********

what does this mean?). If published, this will include your full peer review and any attached files.

If you choose “no”, your identity will remain anonymous but your review may still be made public.

**Do you want your identity to be public for this peer review?**  For information about this choice, including consent withdrawal, please see our Privacy Policy

Reviewer #1: No

Reviewer #2: **Yes:**  Alan Douglas de Lima Rocha

**********

[NOTE: If reviewer comments were submitted as an attachment file, they will be attached to this email and accessible via the submission site. Please log into your account, locate the manuscript record, and check for the action link "View Attachments". If this link does not appear, there are no attachment files.]

To ensure your figures meet our technical requirements, please review our figure guidelines: https://journals.plos.org/plosone/s/figures

You may also use PLOS’s free figure tool, NAAS, to help you prepare publication quality figures: https://journals.plos.org/plosone/s/figures#loc-tools-for-figure-preparation.

NAAS will assess whether your figures meet our technical requirements by comparing each figure against our figure specifications.

# Author comment: Author response to Decision Letter 2

**DOI:** 10.1371/journal.pone.0362259.r004

11 Sep 2026

Dear Academic Editor (Dr. Daniel F. M. Monte) and Reviewer #1,Thank you for your constructive guidance during this second round of review. We have addressed every comment raised by Reviewer #1. A complete, formatted 'Response to Reviewers' PDF has also been uploaded separately to the submission system.Below is our point-by-point response:Responses to Reviewer #1

Comment 1: Choice of Comparator Sequence (K-12 vs. Prototypical UPEC References)Response: We thank the reviewer for this insightful critique. We expanded our comparative alignment by retrieving and incorporating full-length emrD sequences from two prototypical UPEC reference strains—CFT073 (NCBI Accession: AAN83032.1) and 536 (NCBI Accession: WP_001335494.1)—alongside K-12 MG1655 across our 10 clinical isolates. This dual-comparator approach allowed us to methodologically categorize substitutions into baseline UPEC lineage-conserved features (V170A and V304I) and novel, isolate-specific variants (A173T in isolate DUH 02). We updated the Materials and methods, Results, Discussion, Limitations, and Conclusion sections, as well as Table 2 and the S2 figure legend, to reflect this expanded dataset.

Comment 2: Diversity of the 25 BLAST SequencesResponse: We updated our primary sequence processing workflow to directly include CFT073 and 536 as alignment controls. We also clarified in the In Silico conservation and frequency analysis subsection that the 25 BLASTp sequences represent a curated, non-redundant global panel used specifically to calculate substitution frequencies across diverse pathotypes and geographic origins without overcrowding the core alignment.

Comment 3: Methodological Reporting Gaps (PCR Controls, UPEC Definition, MLST, and Sequence Coverage)Response: We addressed all four reporting details:PCR Controls: Explicitly stated that negative non-template controls (NTC; nuclease-free water) were routinely included in every PCR batch to rule out contamination.UPEC Definition: Clarified that isolates were operationally defined using clinical diagnostic criteria (105 CFU/mL pure culture from symptomatic UTI patients).Phylotyping/MLST: Refined the framing of isolate DUH 11 to avoid speculative claims about specific sequence types or phylogroups without MLST data.Sequence Coverage: Updated sequence descriptions and the Limitations section to explicitly report the analyzed coverage range (residues 32–345), noting that N-terminal (~31 aa) and C-terminal (~50 aa) primer quality trimming left terminal ends uncharacterized.

Comment 4: Conceptual Framing & Biofilm/ESBL UncouplingResponse: We updated the subsection title to "Co-occurrence of EmrD variants and beta-lactamase genes" to ensure it accurately reflects our findings without implying empirical biofilm testing. Additionally, we explicitly clarified in the Discussion that the plasmid-borne ESBL profile of isolate DUH 11 is uncoupled from its chromosomal emrD sequence divergence.

Attachment Submitted filename: Response_to_Reviewers_auresp_2.docx

# Peer review: Decision Letter 2

**DOI:** 10.1371/journal.pone.0362259.r005

**Authors:** Daniel F. M. Monte

22 Oct 2026

Molecular diversity and structural profiling of the EmrD efflux pump in multidrug-resistant uropathogenic Escherichia coli.

PONE-D-26-16505R2

Dear Dr. Alnaqshabandy,

We’re pleased to inform you that your manuscript has been judged scientifically suitable for publication and will be formally accepted for publication once it meets all outstanding technical requirements.

Within one week, you’ll receive an e-mail detailing the required amendments. When these have been addressed, you’ll receive a formal acceptance letter and your manuscript will be scheduled for publication.

An invoice will be generated when your article is formally accepted. Please note, if your institution has a publishing partnership with PLOS and your article meets the relevant criteria, all or part of your publication costs will be covered. Please make sure your user information is up-to-date by logging into Editorial Manager at Editorial Manager® and clicking the ‘Update My Information' link at the top of the page. For questions related to billing, please contact billing support.

If your institution or institutions have a press office, please notify them about your upcoming paper to help maximize its impact. If they’ll be preparing press materials, please inform our press team as soon as possible -- no later than 48 hours after receiving the formal acceptance. Your manuscript will remain under strict press embargo until 2 pm Eastern Time on the date of publication. For more information, please contact onepress@plos.org.

Kind regards,

Daniel F. M. Monte, Ph.D.

Academic Editor

PLOS One

Additional Editor Comments (optional):

The comments have been addressed and the reviewers are satisfied.

Reviewers' comments:

Reviewer's Responses to Questions

**Comments to the Author**

Reviewer #1: All comments have been addressed

Reviewer #2: All comments have been addressed

**********

2. Is the manuscript technically sound, and do the data support the conclusions?

Reviewer #1: Yes

Reviewer #2: Yes

**********

3. Has the statistical analysis been performed appropriately and rigorously?

Reviewer #1: N/A

Reviewer #2: Yes

**********

4. Have the authors made all data underlying the findings in their manuscript fully available?

The PLOS Data policy

Reviewer #1: Yes

Reviewer #2: Yes

**********

5. Is the manuscript presented in an intelligible fashion and written in standard English?

Reviewer #1: Yes

Reviewer #2: Yes

**********

Reviewer #1: Thank you for addressing my previous comments. The revisions have adequately addressed my concerns, and I have no further major comments.

Reviewer #2: The revisions are clear and satisfactory. I have no further concerns regarding research or publication ethics.

**********

what does this mean?). If published, this will include your full peer review and any attached files.

If you choose “no”, your identity will remain anonymous but your review may still be made public.

**Do you want your identity to be public for this peer review?**  For information about this choice, including consent withdrawal, please see our Privacy Policy

Reviewer #1: No

Reviewer #2: **Yes:**  ALAN DOUGLAS DE LIMA ROCHA

**********

# Editor report: Acceptance letter

**DOI:** 10.1371/journal.pone.0362259.r006

**Authors:** Daniel F. M. Monte

PONE-D-26-16505R2

PLOS One

Dear Dr. Alnaqshabandy,

I'm pleased to inform you that your manuscript has been deemed suitable for publication in PLOS One. Congratulations! Your manuscript is now being handed over to our production team.

At this stage, our production department will prepare your paper for publication. This includes ensuring the following:

* All references, tables, and figures are properly cited

* All relevant supporting information is included in the manuscript submission,

* There are no issues that prevent the paper from being properly typeset

You will receive further instructions from the production team, including instructions on how to review your proof when it is ready. Please keep in mind that we are working through a large volume of accepted articles, so please give us a few days to review your paper and let you know the next and final steps.

Lastly, if your institution or institutions have a press office, please let them know about your upcoming paper now to help maximize its impact. If they'll be preparing press materials, please inform our press team within the next 48 hours. Your manuscript will remain under strict press embargo until 2 pm Eastern Time on the date of publication. For more information, please contact onepress@plos.org.

You will receive an invoice from PLOS for your publication fee after your manuscript has reached the completed accept phase. If you receive an email requesting payment before acceptance or for any other service, this may be a phishing scheme. Learn how to identify phishing emails and protect your accounts at https://explore.plos.org/phishing.

If we can help with anything else, please email us at customercare@plos.org.

Thank you for submitting your work to PLOS One and supporting open access.

Kind regards,

PLOS One Editorial Office Staff

on behalf of

Dr. Daniel F. M. Monte

Academic Editor

PLOS One
